# Assessment of ground deformation and seismicity in two areas of intense hydrocarbon production in the Argentinian Patagonia

**DOI:** 10.1038/s41598-022-23160-6

**Published:** 2022-11-10

**Authors:** Guillermo Tamburini-Beliveau, Javier A. Grosso-Heredia, Marta Béjar-Pizarro, Raúl Pérez-López, Juan Portela, Martín Cismondi-Duarte, Oriol Monserrat

**Affiliations:** 1 Centro de Investigaciones y Transferencia de Santa Cruz (CIT SC - CONICET), Lisandro de la Torre 860, Río Gallegos, Argentina; 2 Departamento de Geografía, Universidad del Comahue, Buenos Aires 1400, Neuquén, Argentina; 3 Instituto Geológico y Minero de España (IGME-CSIC), Rios Rosas, 23, 28003 Madrid, Spain; 4 Universidad Politécnica de Madrid. GI-Terra: Geomática, Amenazas Naturales y Riesgos, Mercator 2, 28031 Madrid, Spain; 5 Instituto de Investigación y Desarrollo en Ingeniería de Procesos y Química Aplicada (IPQA-CONICET-UNC), Ciudad Universitaria, Córdoba, Argentina; 6 Departament de Geomàtica, Centre Tecnològic de Telecomunicacions de Catalunya, Carl Friedrich Gauss 7, Castelldefels, Spain

**Keywords:** Environmental impact, Seismology, Environmental sciences

## Abstract

The exploitation of both conventional and unconventional hydrocarbons may lead to still not well-known environmental consequences such as ground deformation and induced/triggered seismicity. Identifying and characterizing these effects is fundamental for prevention or mitigation purposes, especially when they impact populated areas. Two case studies of such effects on hydrocarbon-producing basins in Argentina, the Neuquén and the Golfo de San Jorge, are presented in this work. The intense hydrocarbon production activities in recent years and their potential link with the occurrence of two earthquakes of magnitude 4.9 and 5 near the operating well fields is assessed. A joint analysis of satellite radar interferometry and records of fluid injection and extraction demonstrate that, between 2017 and 2020, vertical ground displacements occurred in both study areas over active well fields that might indicate a correlation to hydrocarbon production activities. Coseismic deformation models of the two earthquakes constrain source depths to less than 2 km. The absence of seismicity before the beginning of the hydrocarbon activities in both areas, and the occurrence of the two largest and shallow earthquakes in the vicinity of the active well fields just after intensive production periods, points towards the potential association between both phenomena.

## Introduction

Hydrocarbons are a highly demanded raw material and the exploitation of basement reservoirs for hydrocarbon production is a fundamental industrial activity. However, the economic and social benefits of its extraction are often accompanied by undesirable consequences such as environmental impacts and social unrest. The anticipation to these collateral effects is key to ensure the good performance of the hydrocarbon production activities.

The literature illustrates various examples of geohazards triggered by the hydrocarbon industry around the globe such as ground displacements associated with fluid injection and extraction^1–3^; seismicity induced by fluid extraction and associated surface deformation (e.g. Lacq gas field in France^4^, the Groningen field in the Netherlands^5^); seismicity correlated to the injection of wastewater and enhanced oil recovery^6^, to shale gas hydraulic fracturing^7–9^, and to hydraulic stimulation for enhanced geothermal systems, which operates in a similar way to hydrocarbon production^10^. Maximum magnitudes of earthquakes triggered by these activities can reach significant magnitudes, as occurred in Oklahoma in November 2011, with a Mw 5.7 earthquake related to wastewater injection^11^ or in Mexico in 2011, with a Mw 7.2 earthquake related to fluid extraction at a geothermal field^12^.

**Figure 1:**
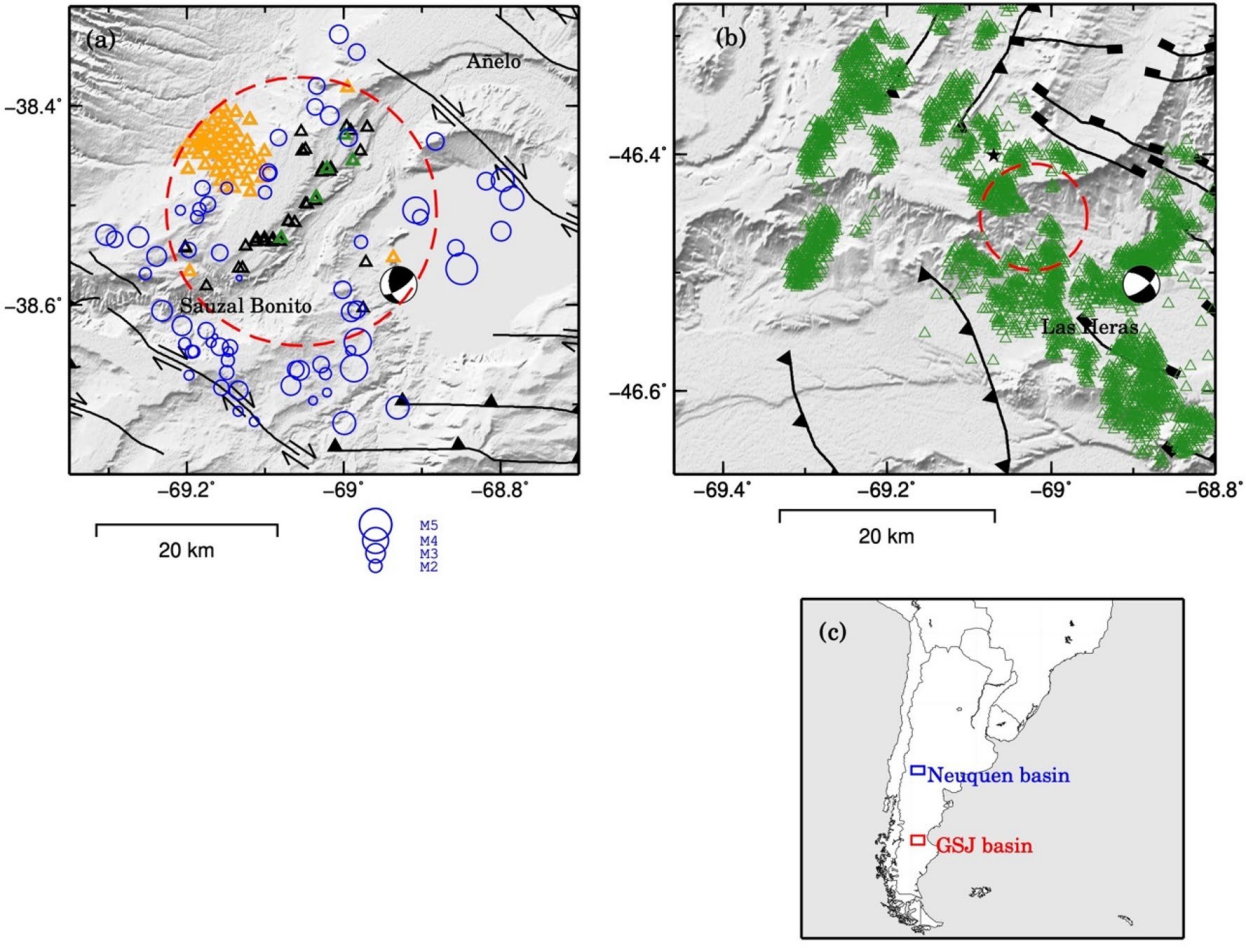
Study areas in the Argentinian Patagonia. Data are plotted on SRTM topography^18^ displayed in shaded relief. (**a**) Neuquén basin area. Blue circles represent epicentres of seismic events scaled by local magnitude (ML) occurred in the period November 2015 to November 2020, from the Instituto Nacional de Prevención Sísmica (INPRES) catalog. The red dashed circle indicates the 15 km radius area analysed in Fig. 3a. Black triangles indicate the location of fracking wells (where unconventional hydrocarbons are extracted after slickwater injection), orange triangles indicate the location of conventional wells and green triangles indicate the location of wastewater injection wells (locations from the Argentine Energy Secretariat). Black lines indicate the main faults from^19^. The Global Centroid Moment Tensor (GCMT, www.globalcmt.org/) focal mechanism of the 07/03/2019 earthquake (ML 4.9, Mw 5) is shown. (**b**) Golfo de San Jorge area. Green triangles represent production wells. Black lines indicate the main faults^20^. The red dashed circle indicates the 5 km radius area analysed in Fig. 7a. The Global Centroid Moment Tensor (GCMT) focal mechanism of the 17/10/2019 earthquake (ML 5, Mw 4.9) is shown. (**c**) Inset map showing the region of South America with the two study areas delineated in blue (Neuquén basin) and red (GSJ basin).

Three of the five hydrocarbon-producing basins of Argentina are located in Patagonia. The Neuquén basin in North Patagonia was one of the first exploitations in Argentina. The operations started at the beginning of the twentieth century continuing up to the present date. The unconventional^13^, 20 exploitations of tight and shale geological formations in the basin started in 2011. The estimated unconventional reserves of the Neuquén basin are 9000 billion of natural gas m^3^ and 2.5 billion of oil m^3^. According to the US Energy Administration, these formations are the second biggest of shale gas and the fourth of shale oil at global scale^14^. The initiation of the hydraulic fracturing in the Vaca Muerta formation, a well-known shale formation in the Neuquén basin, has been followed by a significant increase of the seismic activity, see Fig. 1a.

In South Patagonia, the Golfo de San Jorge (GSJ) basin is also a historical hydrocarbon-producing basin, where more than forty thousand wells have been drilled since the first perforations started searching for water in this arid region. Unlike the Neuquén basin, only conventional^13^ resources are exploited here (Fig. 1b).

Despite the intensive hydrocarbon activity carried out in these basins, a lack of comprehensive analysis on the risk of triggering geohazards such as ground deformation and induced seismicity is obvious. Only two publications addressed the topic in the Neuquén basin^15,16^ and none in the GSJ basin. The recent occurrence of two significant earthquakes within the hydrocarbon production fields in these two basins, the M_L_ 5 2019 October 17 earthquake, near the town of Las Heras in the GSJ basin, and the M_L_ 4.9 2019 March 7 event, near the village of Sauzal Bonito in the Neuquén basin (Fig. 1), have caused social unrest among the population. It is worth noting that these areas are located in the extra-Andean low seismic region^17^, and that no previous earthquakes had been reported prior to the beginning of the hydrocarbon production activities.

This study aims to analyse the relationship between hydrocarbon activities and ground displacements and the start of seismic events in two areas of Argentinian Patagonia. For this purpose, records of fluid injection and extraction, number of hydraulic fracture stages, seismicity and ground movements measured with Satellite Synthetic Aperture Radar Interferometry (DInSAR) data are jointly analysed for the period January 2017 to December 2020.

## Results

### The Neuquén basin

The available dataset analysed in the Neuquén basin include displacement measurements obtained with Differential Synthetic Aperture radar interferometry (DInSAR) and Small Baseline approach (SBAS), seismic data and monthly volume production of conventional and unconventional wells, including injection volumes. We focused the analysis on a region surrounding the epicentre of the main earthquake (7th of March of 2019), which in the analysed period has experienced an intense hydrocarbon production, a high concentration of seismic events and ground displacements in different areas.

**Figure 2:**
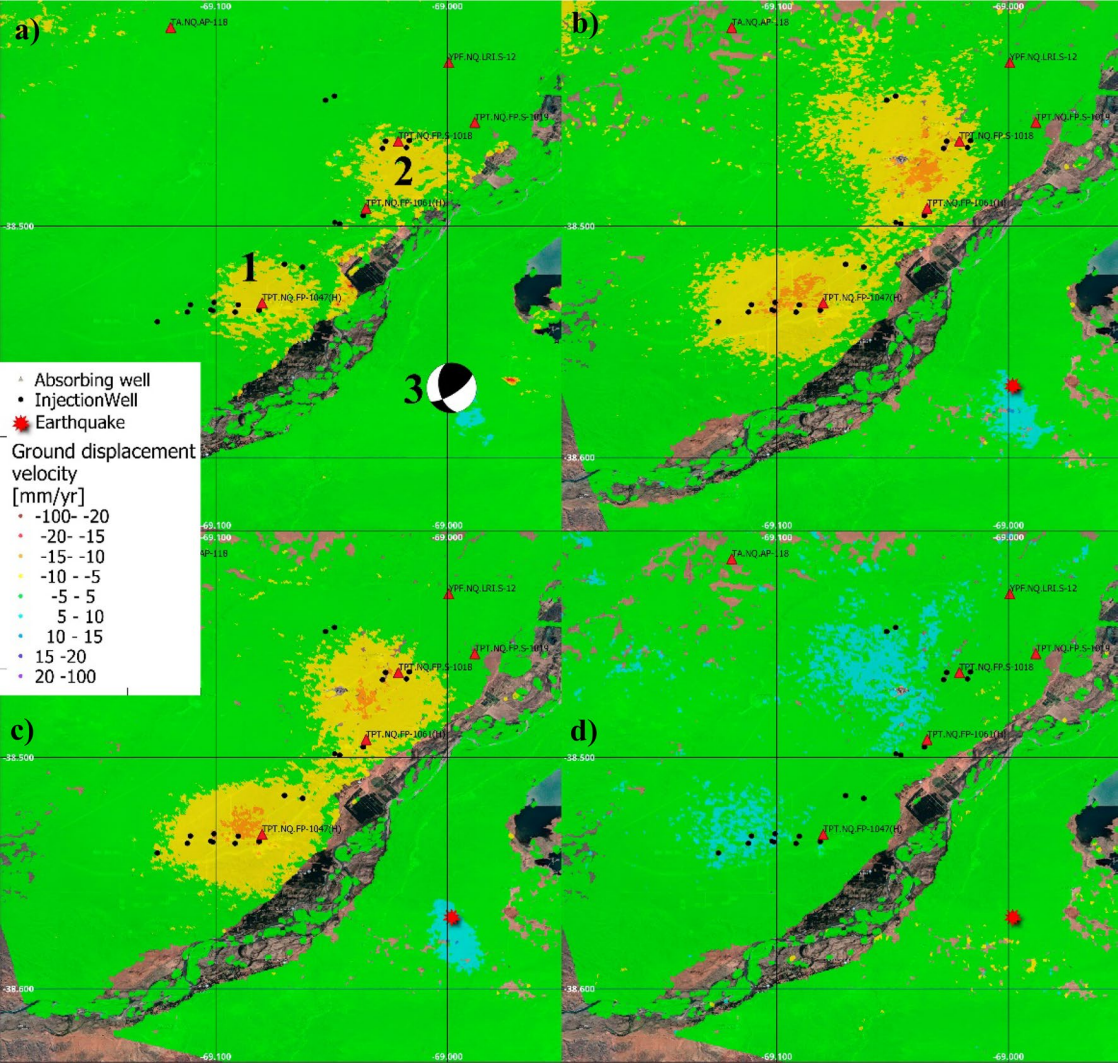
Ground displacement velocity maps obtained from ascending (**a**) and descending trajectory (**b**) superimposed to a Google satellite image. (**c**) and (**d**) Show the ground displacement vertical and West–East component respectively. The analysed period spans from January 2017 to December 2020. Main deformation zones labelled 1 and 2 correspond to areas with high concentration of wells, 33 and 25 respectively. Absorbing Wells (red triangles) indicate the location of wastewater disposal wells. Red star indicates the main area affected by the ML 4.9, 2019 March 7 earthquake and 3 shows its Global Centroid Moment Tensor (GCMT) focal mechanism.

Figure 2 shows the displacement velocity maps obtained through the SBAS analysis, performed with the PSIG software^21,22^, exploiting the ascending and descending trajectories of Sentinel-1 data. The colour scale represents the velocities of displacement of each point in mm/year. Figure 2a and b show the displacements along the satellite’s Line-Of-Sight (LOS), which means that the measurements represent the projection of the real displacements along the satellite-point line. Red tones represent points moving far from the satellite while blue ones show points moving towards the satellite. Figure 2c and d show the vertical and west–east components of the displacement. These components are derived from the LOS results following the approach described in^23^. Red tones represent movements down and towards the east in the vertical and horizontal components respectively. It is worth noting the quality of these components rely on the quality of the LOS results. The descending dataset has a period (February 2018 to October 2018) without images. This created some uncertainties on the phase unwrapping which result in residual terms in Vertical and Horizontal components.

The results presented in Fig. 2 allowed the identification of two ellipsoidal shape ground displacement regions, with an approximate radius of 2.3 km. In these areas, identified by the numbers 1 and 2 in Fig. 2, a high number of new fracking wells have been drilled. The analysis of these deformation regions is provided in section “Hydrocarbon production and ground dispolacements in the Neuquén basin”. The SBAS analysis allowed the identification of a third feature, labelled number 3 in Fig. 2, which is related to coseismic deformation caused by the 2019 March 7 earthquake, which is discussed later in this document. The results refer to the periods January 2017 to December 2020 in both trajectories. The precision of the estimated velocities is about ± 1.7 mm/year in the ascending trajectory and ± 2.3 mm/year in the descending one.

**Figure 3:**
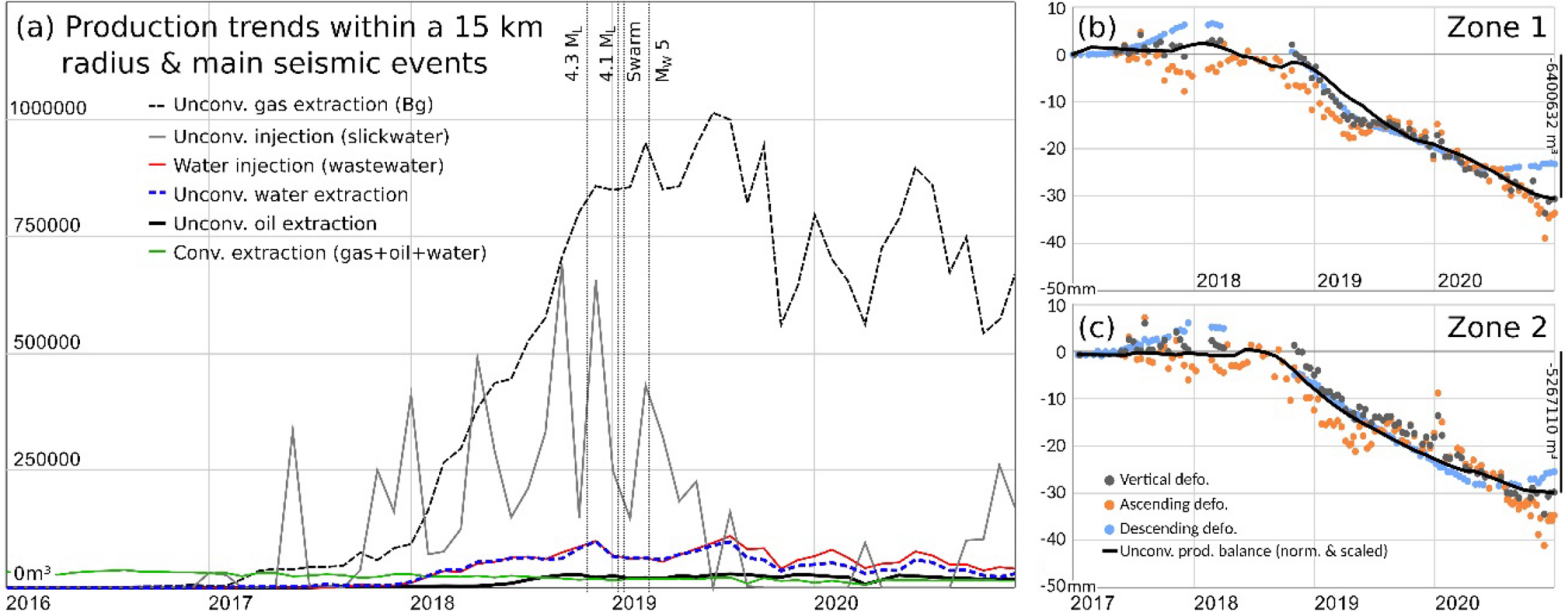
(**a**) Evolution of hydrocarbon production (in m^3^) between 2016 and 2020 in the area bounded by the red dashed circle in Fig. 1a. The vertical dashed lines show the main seismic events. Panels (**b**) and (**c**) show the time series of ground deformation for zones 1 and 2, respectively, during the period 2017–2020 and the monthly accumulated unconventional production balance (black line) normalised and inversely scaled. The vertical scale bar in the right axis of figures (**b**) and (**c**) provide information on the total accumulated volumetric amount (m^3^) of unconventional fluid balance.

Figure 3a shows the harmonised hydrocarbon production data in the Neuquén basin study area during the period 2015–2020: monthly extraction (conventional and unconventional), wastewater injection and fracking injection. The harmonisation procedure is described in the "Methods" section. The main seismic events have also been depicted as vertical lines. Figure 3b and c displays the time series of ground deformation obtained from the ascending and descending trajectories in zones 1 and 2, respectively, together with the curve of the total accumulated unconventional fluid balance for each area. The latter parameter has been standardised (normalised and scaled) to better show the correlation between both processes. The normalisation is described in the "Methods" section.

#### Hydrocarbon production and ground displacements in the Neuquén basin

The deformation velocities revealed ground displacement in zones 1 and 2 (Fig. 2). The deformation time series (TSs) of these areas can be observed in Fig. 3b and c, respectively. These TSs show that the temporal behaviour reflect changes along the monitored period. The ascending and descending TSs show a good agreement in both temporal behaviour and magnitude. For these time series, the estimated precision is 2.8 mm and 2.0 mm for ascending and descending trajectories respectively. Figure 3b and c shows also the estimated vertical component for the same area. The good agreement between Ascending and Descending trajectories may be explained by a movement almost vertical, and, by a subsidence. This is reflected by the vertical component time series.

The analysed time series can be decomposed in four periods. Almost quiescence, from January 2017 to September 2018, period of linear movement, from September 2018 to May 2019 corresponding with the increase of extraction activities, a soft decrease of the movement rates up to October 2019 and again, a period of linear movement lasting up to December 2020. The acceleration and deceleration periods are in good agreement with the reduction and increase of extraction activities, see Fig. 3b,c.

Figure 2c and d confirm that the vertical component is dominant in both areas. One may observe soft horizontal component. However, this component can be explained by residual errors triggered by the void of images of the descending dataset during the year 2018. The average accumulated vertical displacements for the areas 1 and 2 are 33.0 mm and 28.2 mm respectively. The average velocity in zone 1 is − 9.6 mm/year in the ascending trajectory and − 8.4 mm/year in the descending one. The maximum velocities of deformation reaches 13 mm/year in LOS in the centre of the ellipsoidal areas. The differences in velocity between both trajectories are related to the estimated precision. Similar figures are obtained for zone 2. The comparison between both trajectories confirms the reliability of the results and confirms that the displacements in zones 1 and 2 are almost vertical and therefore can be interpreted as subsidence.

Figure 3a shows the beginning of unconventional activity in January 2017. A clear change in the trend occurred in July 2017 with the initiation of strong fracking visible in the first high injection peak and reflected in the later increase in the gas extraction (around the beginning of 2018). Zones 1 and 2 are affected by the accumulated action of multiple wells working simultaneously but in different stages of the production chain (fracturing or extracting). Considering the period framed in Fig. 3a, the predominant activity in terms of subsurface volume changes is gas extraction. Therefore, the expected surface displacements triggered in this period is subsidence^4,5,12,24^, which agrees with the observed displacements.

To understand the relationship between ground deformation and hydrocarbon production activities, we modelled ground displacements over zones 1 and 2 using a simple Mogi approach (see "Methods" and supplementary figures S4, S5, S6 and Table S1 for details on the inversion procedure). We inverted ground deformation accumulated in the area during the complete period (January 2017–December 2021). The deformation pattern observed over zones 1 and 2 can be explained with two Mogi sources located at 4.5 km depth (source south) and 3.4 km depth (source north). These depths are consistent with the average depth of wells operating in both areas (3.3 km depth and 3.4 km depth in the southern and northern area respectively, according to the Argentine Energy Secretariat databases). The volume change of the optimal model is − 2.7E + 06 m^3^ for the southern Mogi source and − 1.54E + 06 m^3^ for the northern Mogi source. The total volume changes of extracted and injected fluids (gas, oil, water, wastewater, slikwater) in the wells operating in both areas during the same period are higher: − 6.4E + 06 m^3^ in the south and − 5.3E + 06 m^3^ in the north (Argentine Energy Secretariat databases). These values are the same order of magnitude than the Mogi results, and are also negative volume changes, which is consistent with dominating fluid extraction during the study period.

#### Hydrocarbon production and seismicity in the Neuquén basin

The analysis of multiple source seismic catalogues^25–27^ reveals that no earthquakes were registered in the study area before 2015. This quiescence suddenly changed with the intensification of hydraulic fracturing activities in the area^15,16^, as shown in Fig. 1. Therefore, we investigate the relationship between the hydrocarbon activities and the observed seismicity.

Seismic sequences potentially classified as induced seismicity normally show a temporal and spatial relationship with the underground injection and industrial operations^6,8,28^. To investigate the potential relationship between seismicity and hydrocarbon activities in the study area, the seismic events recorded by the National Institute of Seismic Prevention of Argentina (Instituto Nacional de Prevención Sísmica, INPRES^29^) during the period November 2015 to November 2020 are analysed (supplementary figures S1, S2). A total of 63 earthquakes were registered in this period, with a maximum earthquake of local magnitude (M_L_) 4.9 (at 7 km depth), and a magnitude of completeness (Mc) 2.4 (supplementary figure S3). The analyses of earthquake statistical basic parameters (the b-value of the Gutenberg-Richter law, the maximum earthquake, magnitude of completeness and hydraulic depth operations and horizontal longitude) are included in the Figure S3 of the supplementary information of this article. The b-value obtained is 0.66 (correlation coefficient of 0.94). However, the number of earthquakes is not enough to determine the significance of this value in relation to other bibliographic values^30^.

**Figure 4:**
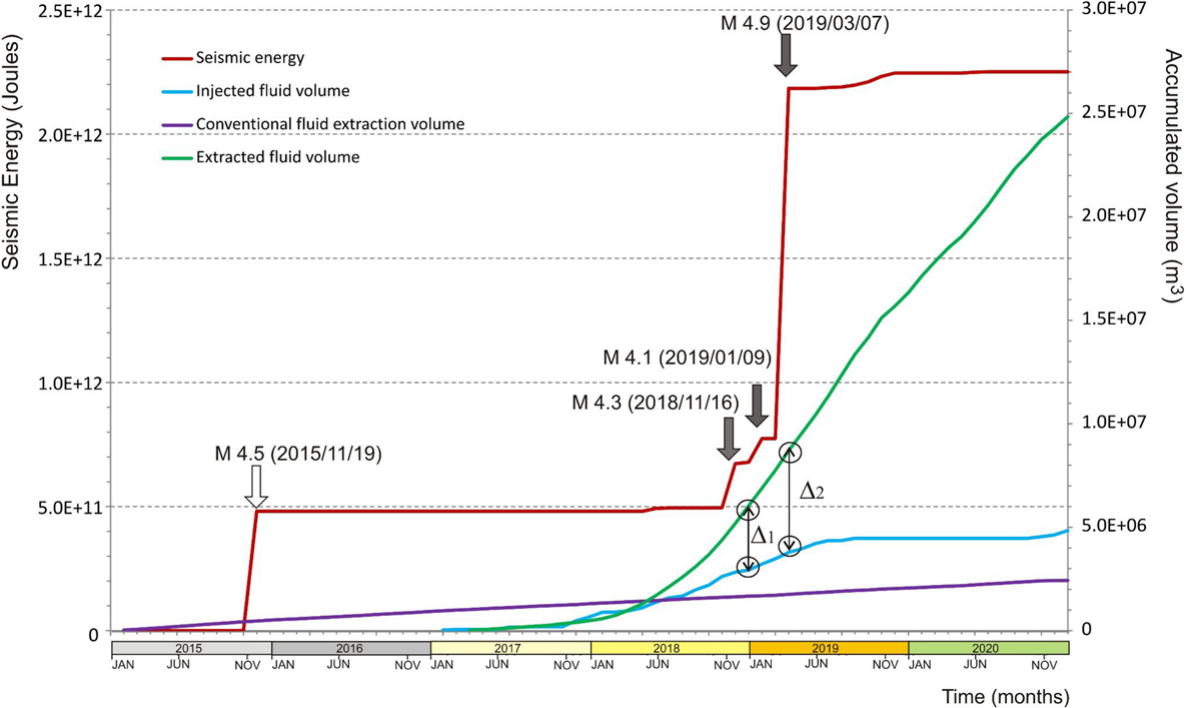
Accumulated seismic energy released (in Joules) recorded by INPRES between 2015 and 2020, versus different underground operations within the Neuquén basin study area (in m^3^). Blue and green lines show injected and extracted fluid volume, respectively, using unconventional methods. Purple line shows extracted fluid volume using conventional methods. The production data used for this figure are the same used in Fig. 3.

The temporal relationship between fracking underground operations and earthquake occurrence, for the period January 2015 to December 2020, is shown in Fig. 4.

The accumulated seismic energy is compared with accumulated volumes of injection/extraction of fluids during both conventional and unconventional hydrocarbon production activities.

The 2019 earthquakes (M_L_ 4.1 and M_L_ 4.9) appeared at the final stages of fluid injection and at the early stages of fluid extraction. Also, and related to the levelling of fracking injection (blue line), the seismic energy decreased to a low threshold without relevant earthquakes of M_L_ > 3.6. The largest seismic event registered in the study area occurred on 7 March 2019 at 05:10:37 UTC, with local magnitude M_L_4.9, according to INPRES and moment magnitude M_w_ of 5 according to the^26^. The location of the epicentre and depth of the earthquake varies among different agencies (USGS: latitude 38.523° S, longitude 68.857° W, 13.8 km depth; INPRES: latitude 38.563° S, longitude 68.833° W, 7 km depth;^16^: latitude 38.529° S, longitude 68.891° W, 10.3 km depth).

**Figure 5:**
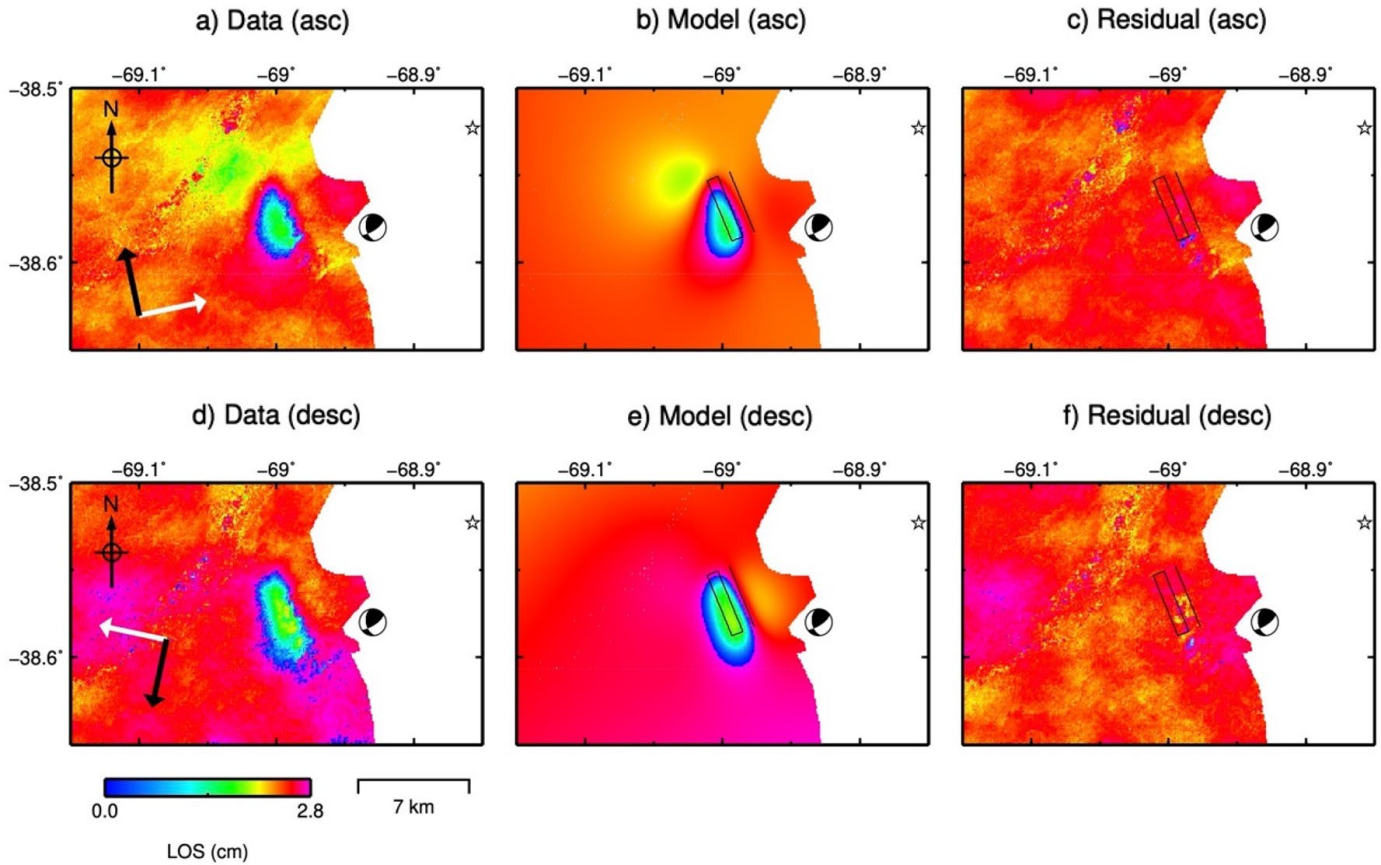
(**a**) and (**d**) Show coseismic Line-Of-Sight (LOS) deformation of the Mw 5, 2019 March 7 Neuquén basin earthquake obtained as the average of 11 coseismic ascending interferograms (in a) and as the average of 10 coseismic descending interferograms (in **d**). The white arrows indicate satellite Line-of-Sight direction (LOS) and the black arrows indicates satellite azimuth (Az). (**b**) and (**e**) Show LOS deformation predicted by the forward model using the maximum a posteriori probability solution. (**c**) and (**f**) show the residuals. The black rectangle represents the outline of the optimal fault plane. The Global Centroid Moment Tensor (GCMT, www.globalcmt.org/) is represented by the beach ball.

This seismic event produced ground deformation measurable with InSAR data. We used the coseismic interferograms to constrain the earthquake source parameters. Figure 5 shows ground deformation associated with the earthquake in both ascending and descending geometry. This deformation pattern corresponds to a main lobe with a maximum ground displacement of ~ 22 mm towards the satellite.

This deformation pattern can be explained using a uniform slip rectangular dislocation centred at latitude 38.5691° S, longitude 68.9982° W (Fig. 5) and at 1.2 km depth. The fault plane is consistent with the SW dipping plane of the Global Centroid Moment Tensor solution. The geodetic moment corresponding to our optimal solution is 2.38e + 16 N m, which is consistent with the GCMT seismic moment (2.19e + 16 N m). See "Methods" and supplementary material (figures S7, S8 and Table S2) for more details on the inversion procedure.

The epicentres estimated by other agencies are located 11 to 17 km north/northeast of the location of our preferred model (beneath Los Barreales Reservoir, see supplementary Figure S9 and S10) and the hypocentres are 5.8–12.6 km deeper. These locations are not compatible with the measured ground deformation. This difference can be explained because the Vaca Muerta region is deficiently covered by a seismic network, with few seismographs located hundreds of kilometres away (see supplementary Fig S1).

The fault plane obtained in the inversion (strike N158° E, dip 56°, rake 37, thrust slip 0.08 m, left-lateral slip 0.11 m) is consistent with the prevailing stress regime in the study area, which can be represented by the first-order horizontal stress (S_Hmax_) obtained from the analysis of focal mechanism solutions (see Supplementary figures S10, S11, S12). S_Hmax_ is oriented N112°-trend, which agrees with the local stress orientation obtained by^31^ and the local/regional stress obtained by^32^ (see Supplementary Table S3).

### The Golfo de San Jorge (GSJ) basin

This section follows an analogue structure as the previous one. The available data for the study of the GSJ basin include displacement measurements obtained with InSAR methods, monthly volumes of conventional wells production and seismic data (a single event). In this basin, there is no relevant presence of unconventional wells.

**Figure 6:**
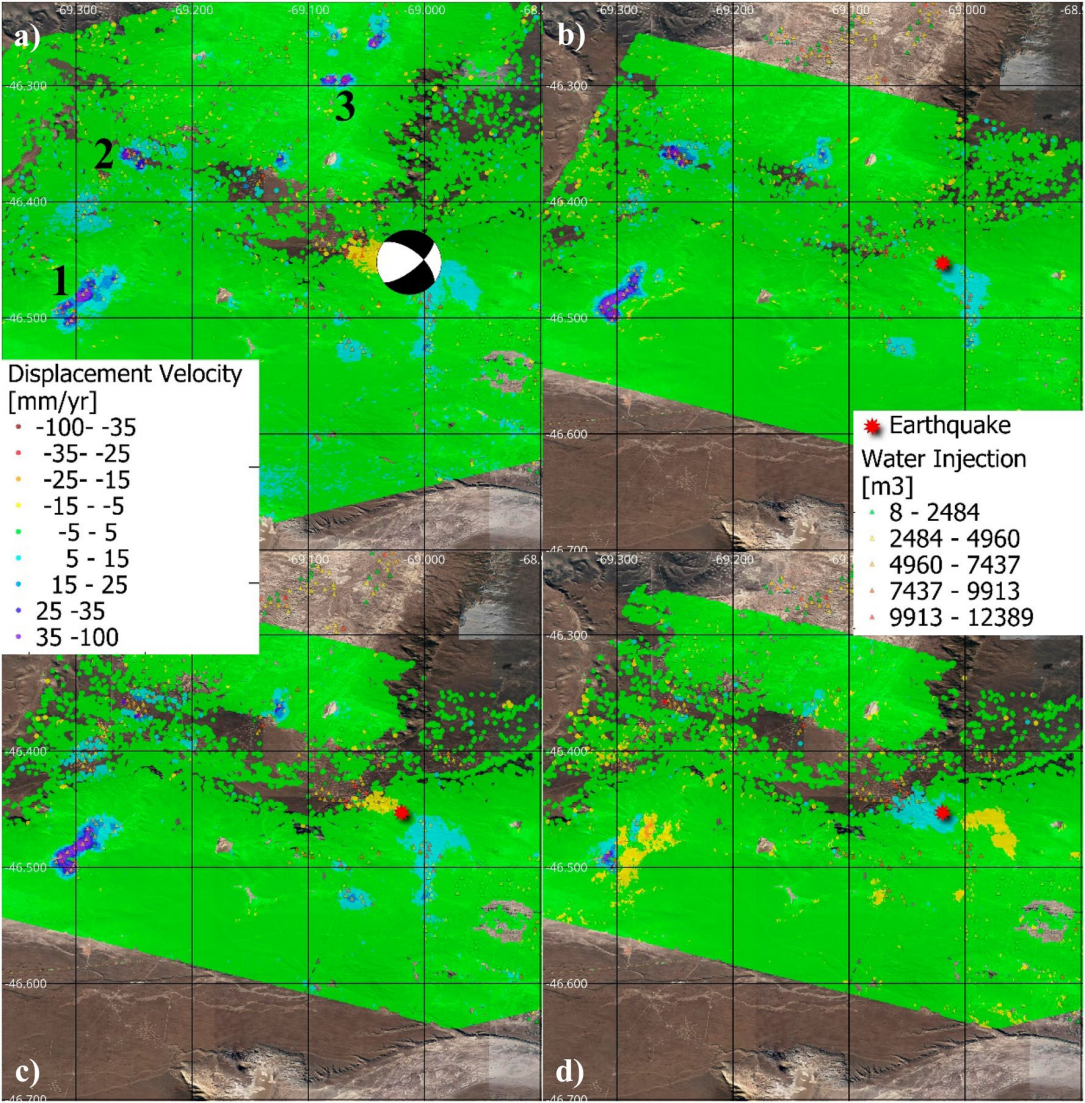
Ground displacement velocity maps obtained exploiting the SBAS technique observed from ascending trajectory (**a**) and descending trajectory (**b**) in the GSJ basin superimposed to a Google satellite image (**c**) and (**d**) show the ground displacement vertical and West–East component respectively. The analysed period spans from January 2017 to December 2020. Positive LOS values are movements towards the satellite. (1, 2, 3) point the areas with higher rates. The red star indicates the location of the ML 5 2019 October 17 earthquake epicentre. (**a**) Shows The Global Centroid Moment Tensor (GCMT) focal mechanism of this earthquake.

Figure 6 shows displacement velocity maps obtained using the Small Baseline approach (SBAS) implemented by CTTC^22^. Figure 6a has been obtained from 108 Sentinel-1 SLC-IW images acquired in descending trajectory covering the period from January 2017 to December 2020. Figure 6b has been obtained from 95 Sentinel-1 SLC-IW images acquired in ascending trajectory during the same period. The colour scale represents the displacement velocity of each point in mm/year in LOS (a and b) and in vertical and horizontal components in c and d. The estimated precision is ± 2.9 mm/year and ± 2.6 mm/year for the descending and ascending datasets, respectively. Time series precision is estimated as 4.3 and 2.1 mm for the ascending and descending respectively.

For this study, areas of ground displacement identified in Fig. 6 as zones 1, 2 and 3 and the coseismic deformation marked with a star are analysed in detail.

#### Hydrocarbon production and ground displacements in the GSJ basin

The displacement rates measured using the ascending and descending trajectories show good agreement in the analysed moving areas (Fig. 6). We can notice some differences that can be explained through its decomposition in horizontal and vertical components. A clear example is observed in zone 1, where the east flank and in particular north-east corner shows a clear blue sector in the ascending result which is less visible in the descending dataset. In this particular case, the difference can be explained by a horizontal component of the movement explained by the local topography of the area which is south-west oriented. There is also a residual error due to phase jump in the ascending dataset triggered by a long period without acquisitions.

**Figure 7:**
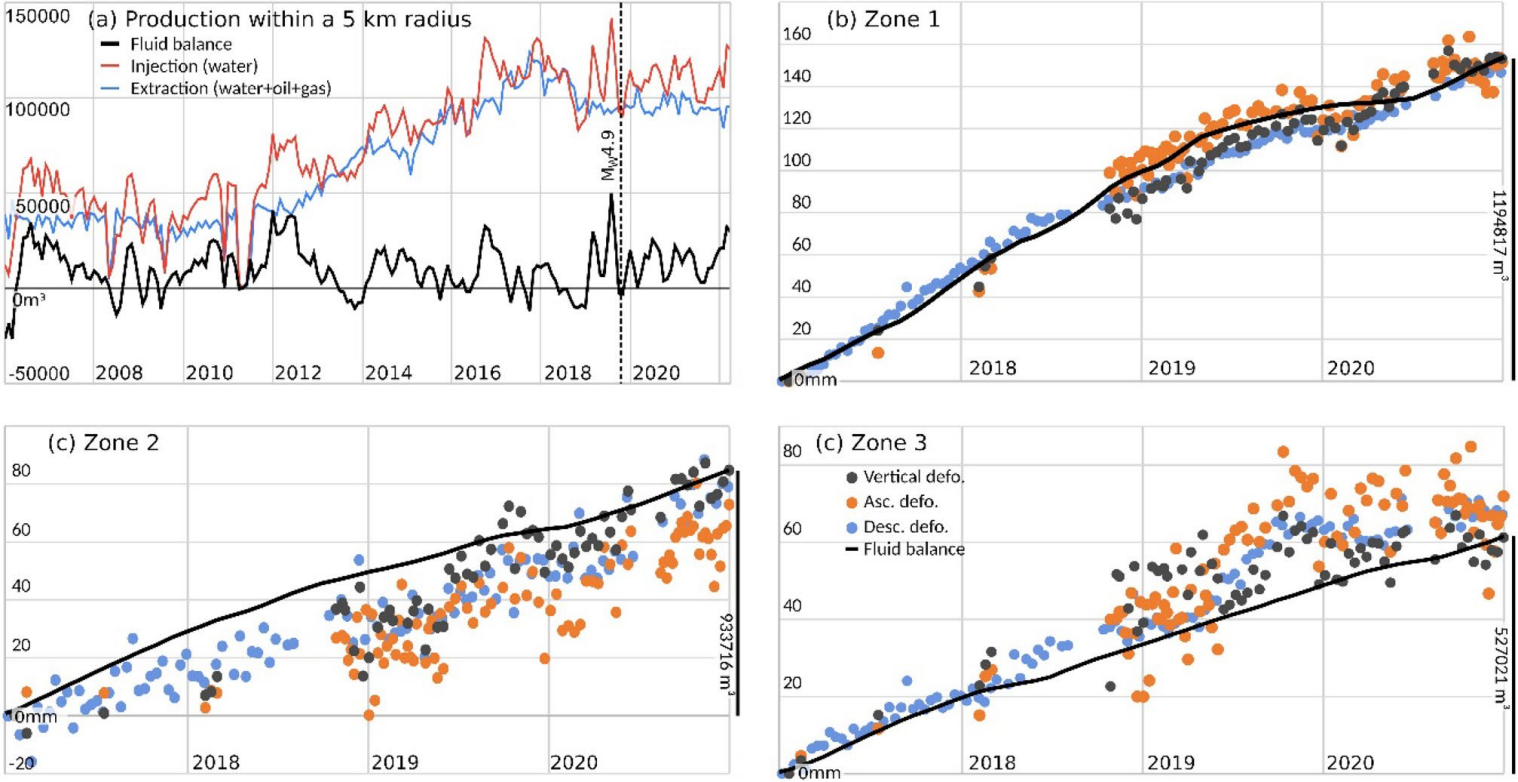
(**a**) Production trends for the last 15 years in an area of 5 km radius (indicated by the red dashed circle in Fig. 1b) that includes the 223 wells (with an average depth of 1.5 km) located around the 2019 October 17 earthquake epicentre (red star in Fig. 6) in the GSJ basin. Fluid (water and hydrocarbons) extraction (blue) and injection (water, red) and balance between them (injection minus extraction, black). The date of the occurrence of the earthquake is indicated by a vertical line in the chronological axis. The earthquake occurs immediately after the highest injection historic peak and in the strongest and sudden historical imbalance. (**b**–**d**) Plots of time-series of deformation (orange for ascending orbit, blue for descending orbit) and standardised fluid balance (black line) for wells over the deformation zones labelled 1,2,3 in Fig. 6a. (**b**) Represents the trends of 85 recovery wells inside deformation zone 1, (**c**) 29 wells in zone 2, and (**d**) 15 wells in zone 3. Besides the standardisation, the scale bar in the right vertical axis provides the magnitude of the accumulated fluid balance during the analised period for the whole deformation zone.

The average displacement time series estimated for zones 1, 2 and 3 using the ascending, descending and vertical trajectories (orange, blue and gray dots, respectively) are shown in Fig. 7b–d. They display similar magnitudes for the ascending and descending trajectories in the areas with maximum movement. Thus, according to these observations, we can conclude that the displacements are almost vertical and hence, uplifts. This is also confirmed by the horizontal and vertical displacement maps (Fig. 6c,d). As commented above, Zone 1 shows a noticeable horizontal component explained by local topography and phase jumps.

The black lines of Fig. 7b–d represent the standardised fluid balance for each area, including the production trends of all the wells within the ground deformation areas revealed by the SBAS result. By fluid balance we refer to injected water minus extracted fluids, both for water and hydrocarbons.

Similarly, to the Neuquén basin deformation case, a Mogi modeling approach can be used to investigate the relationship between ground displacements and hydrocarbon production. However, in the GSJ case, the deformation patterns are more complex: while in the Neuquén basin, ground deformation was mainly concentrated in two areas that are circular, in the GSJ basin, ground deformation is distributed in numerous irregular areas, and thus not easily modeled using simple Mogi sources. We chose zone 3 because it presents a relatively simple deformation pattern. The ground deformation observed over this area during the study period can be explained with two Mogi sources (figure S15) located at 349.1 m depth (source West) and 337.5 m depth (source East).

These depths are consistent with the average depth of wells operating in both areas (642 m and 628 m depth in the western and eastern area respectively, according to the Argentine Energy Secretariat databases).

The volume change of the optimal model is 152.992 m^3^ for the western Mogi source and 125.570 m^3^ for the eastern Mogi source. The total volume changes of extracted and injected fluids (gas and water) in the wells operating in both areas during the same period are higher: 233.415 m^3^ in the west and 283.754 m^3^ in the east. These values are the same order of magnitude than the Mogi results, and are also positive volume changes, which is consistent with dominating fluid injection in both areas during the study period. See "Methods" and supplementary materials (figures S13, S14 and Table S4) for more details on the inversion procedure.

#### Hydrocarbon production and seismicity in the GSJ basin

No earthquakes were registered in the GSJ basin before October 2019 according to the consulted seismic catalogs, including the national seismic catalog INPRES (supplementary figure S1, magnitude of completeness 2.4) and the USGS ANSS Comprehensive Earthquake Catalog^33^. The regional tectonic context is characterized by the presence of contractional structures N-S oriented and normal faults with E–W and NW–SE strikes^19,33^. Although this area is considered aseismic nowadays, analysis of Paleocene normal faults suggest they can generate earthquakes of magnitude greater than 5^35^.

The only seismic event registered in the study area to date occurred on 17 October 2019 at 16:58:28.55 UTC, with a local magnitude of M_L_5 according to INPRES and moment magnitude (M_w_) of 4.9 and 10 km depth according to the USGS^26^. The location of the epicentre and depth of the earthquake varies among different agencies (USGS: latitude 46.401° S, longitude 69.070° W, 10 km depth; INPRES: latitude 46.284° S, longitude 68.734° W, 15 km depth; ISC: latitude 46.421° S, longitude 69.043° W, 10 km depths).

**Figure 8:**
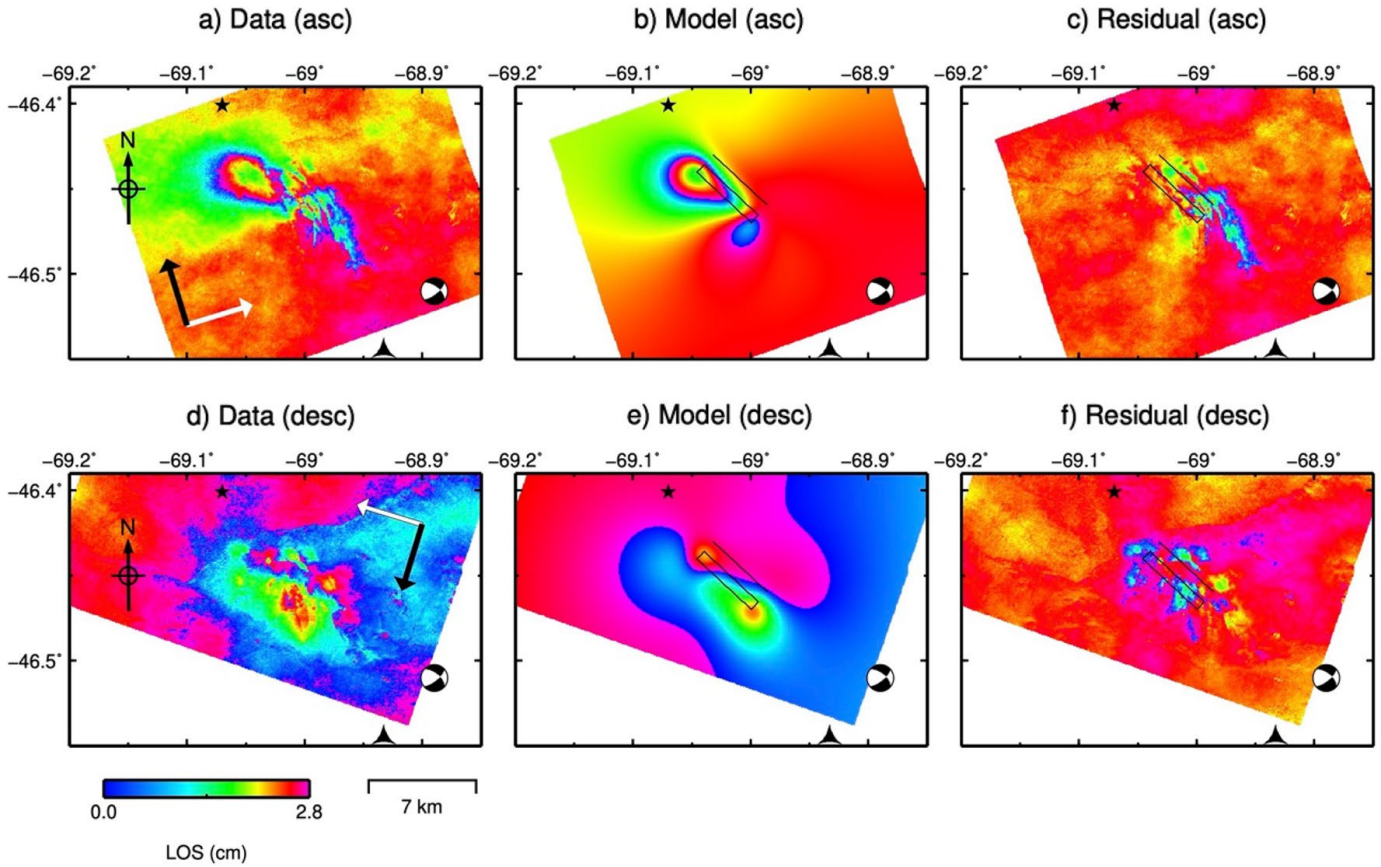
(**a**) and (**d**) Show co-seismic Line-Of-Sight (LOS) deformation of the Mw 4.9, 2019 October 17 GSJ basin earthquake obtained as the average of 4 and 6 co-seismic ascending and descending interferograms, respectively. The white arrows indicate satellite Line-of-Sight direction (LOS) and the black arrows indicates satellite azimuth (Az). (**b**) and (**e**) Show LOS deformation predicted by the forward model using the maximum a posteriori probability solution. (**c**) and (**f**) Show the residuals. The black rectangle represents the outline of the optimal fault plane, and the beach ball represents the fault plane solution from The Global Centroid Moment Tensor (GCMT, www.globalcmt.org/).

This seismic event induced ground deformation measurable with InSAR data. We have used the information provided by coseismic interferograms to constrain the earthquake source parameters. Figure 8 shows ground deformation associated with the earthquake in both ascending and descending geometry. This deformation pattern corresponds to a main lobe with a maximum ground displacement of ~ 35 mm towards the satellite and ~ 40 mm away from the satellite. This deformation pattern can be explained using a uniform slip rectangular dislocation (Fig. 8) centred at latitude − 46.4529° S, longitude 69.0196° W and at 1.17 km depth. The inversion allows to locate the source of the event. The fault plane is consistent with the SW dipping plane of the Global Centroid Moment Tensor solution. The geodetic moment corresponding to our optimal solution (5.28e + 16 N m) is higher than the GCMT seismic moment (2.32e + 16 N m), which might be explained by the presence of post-seismic deformation in the co-seismic interferograms (since they span a post-seismic period of 43 days) or errors in the geodetic and/or seismic model. See "Methods" and supplementary materials (figures S16, S17 and Table S5) for more details on the inversion procedure.

Similar to the Neuquén case, the epicentres of the GSJ 17 October 2019 earthquake estimated by other agencies are located at significant distances from the location of our preferred model (4.5–37 km depending on the agency, see supplementary figure S18) and the hypocentres are deeper (8.8–13.8 km). These locations are not consistent with the measured ground deformation. The different location can be explained also in this case due to the sparse seismic network in the GSJ region (see Figure S1).

The fault plane obtained in the inversion (strike N133° E, dip 53°, rake -10, normal slip 0.05 m, left-lateral slip 0.30 m) is consistent with the prevailing stress regime in the area (the San Bernardo Fold Belt) which is characterized by the presence of contractional structures N-S oriented and normal faults with E–W and NW–SE strikes^20,34^.

## Discussion and conclusions

Two different types of phenomena occurring in two different regions of the Patagonian oilfields, the Neuquén basin and the Golfo de San Jorge basin, have been observed and analysed. For each site, we analysed the relation between surface ground displacements, earthquakes occurred around active well fields and hydrocarbon production operations.

In the Neuquén basin, the focus of the analysis is a zone of intense production of unconventional hydrocarbons, in the region known as Vaca Muerta. We used DInSAR and SBAS techniques to measure the ground displacements of the area. We detected ground displacement due to different sources. Two areas affected by subsidence were detected around two sets of wells. These areas are indicated as 1 and 2 in Fig. 2. The analysis of these areas demonstrated a relationship between well production activities and ground surface displacement. The results obtained show that there was no ground movement during the absence of fracking wells, and that ground movement started a few months after the initiation of fracking operations. Moreover, the collected data about the production activity show a clear imbalance between extraction and injection which is in correspondence with the type of detected movement (subsidence).

Additionally, we investigated the relationship between ground deformation and hydrocarbon production using a Mogi modelling approach. The model obtained explains the deformation pattern observed over zones 1 and 2 with two Mogi sources, consistent to first-order, with the depth and volume changes values estimated from the Argentine Energy Secretariat databases. In both cases, the changes of volume are negative, which is consistent with predominantly fluid extraction during the study period. The differences between the Mogi modelling results and the depths and volumes estimated from the Argentine Energy Secretariat databases can be explained by the assumptions made both by the Mogi modelling and by our volume change estimations. For instance, the volume change obtained in the Mogi modelling refers to the volume change of the cavity; this volume cannot be directly compared with the total change in volume of the extracted and injected fluids, since Mogi assumes an incompressible fluid. On the other hand, the assumption of an isotropic-elastic media in the Mogi models is probably an oversimplification in areas of intense hydraulic fracturing, which can modify the mechanical behavior of the crust. Regarding the volume change estimated from the Argentine Energy Secretariat databases, the conversion of gas volume, from standard to reservoir conditions, although useful to analyze the correlation with other parameters (such as ground deformation) should be regarded as an approximation when considering the total volume change (see "Methods" for more details). These results allows us to confirm the link between wells production and ground subsidence in the studied area.

We also analysed the seismic events occurred during the study period in the Neuquén basin test site. On the one hand, our InSAR analysis revealed a second deformation phenomena related to the M_w_ 5 earthquake that occurred on 7th March 2019. The coseismic interferograms allowed us to precisely locate the source. On the other hand, our analysis of the seismic data and the production history suggests that a correlation exist between the accumulated released seismic energy and the volume changes due to the injection and extraction activities:

- The production data show that in the observed area the hydrocarbon industry started with unconventional wells in 2017 and that a few months later the seismic activity started.
- The data show that, compared to the rest of the analysed period, hydraulic fracturing rates and operated fluid volumes were high immediately prior to the largest seismic events.
- The largest seismic events occurred immediately after the increase of production activities.
- The largest earthquake (M_w_ 5 2019 March 7) shallow depth (1.2 km according to our optimal source model) is consistent with the average depth of wells operating in the study area (~ 3 km).
- Parallelisms between the accumulated released seismic energy and accumulated injection/withdrawal fluids show a good correlation in sharping slope changes. Although this is a qualitative comparison, the correlation shows a temporal coincidence of two a priori independent facts.

As shown in our analysis of seismicity in the Neuquén basin (Fig. 4), a sharp increase of the released seismic energy appears in relationship with the increase of production activities. However, the available data do not allow to conclude which type of production activity (fluid injection or extraction) could be responsible for triggering induced earthquakes.

A similar analysis has been performed in the GSJ area. Although the characteristics of the area are rather different, our analysis allowed us to link ground displacements processes with hydrocarbon operations. As in the Neuquén basin test site, we have studied two types of ground displacements: uplift processes around exploitation wells during the monitored period and a single displacement event related to the M_w_ 4.9 earthquake occurred on 17th October 2019.

We focused the analysis of the uplift phenomena in 3 of the most significant areas, labelled 1, 2 and 3 in Fig. 6. The comparison between uplift phenomena and hydrocarbon exploitation shows that there is a good agreement between the accumulated fluid balance (positive) and the uplift during the measured period, suggesting a cause-effect relationship. We also investigated in this area the relationship between ground deformation and hydrocarbon production using a Mogi modelling approach, focusing on deformation zone 3, which presents a simple deformation pattern distributed in two areas. The ground deformation observed over this area during the study period can be explained by two Mogi sources consistent to first-order with the depth and volume changes values estimated from the Argentine Energy Secretariat databases. In both cases, the changes of volume are positive, which is consistent with predominantly fluid injection during the study period. As in the Neuquén case, the differences between the Mogi models results and the depths and volumes estimated from the Argentine Energy Secretariat databases can be explained by the assumptions made both in the Mogi modelling and for the volume change estimations.

The seismicity in the GSJ study area shows a different behaviour than the Neuquén example. In this case, there is a single event, with no seismicity recorded in the area before or after this event. The highest injection historic peak and the strongest historic imbalance between fluid injection and extraction in this location occurred just some days before the earthquake, see Fig. 7a. The coseismic interferograms allowed us to locate the earthquake at 1.17 km depth, which is consistent with the well depth. The modelled fault plane is consistent with the prevailing stress regime in the area^20,34^. These three facts, shallow earthquake source, absence of record of prior and post seismic events, and the intense injection activity suggest that this event was directly related with the wells operations.

Both cases are two interesting and different examples where the correlation between seismicity and hydrocarbon operations and ground deformation can be clearly shown. Our results also suggest a direct relationship between hydrocarbon production and seismicity in both areas, although additional analyses, such as modelling of induced stress perturbations at the inferred faults^35–38^, and a better characterization of seismic events (e.g. setting a denser seismic network, relocating earthquakes) would help to better understand the relationship between production and induced seismicity.

These case studies represent good examples of how this industry can affect the environment. Moreover, the cases studied in this work affected the population living around the exploitation areas, producing social unrest, from demonstrations at a local scale to legal claims in the National Court^39,40^, that in some cases culminated in the halting of operations. One question that raises up thorough these cases is if those events could be avoided with better characterization of the exploitation sites and with better planning of the injection-extraction activities. These are open questions nowadays but must be addressed by both industry and public administration. This is, for example, especially critical nowadays in Europe, Canada or Brasil, where some potential unconventional exploitation of huge gas reservoirs have been stopped or limited due to the strong opposition of the local population^39,41,42^. Another important point to be considered are the ground displacements that such activities produce and that can potentially produce damages for different reasons, such as infrastructures affectation (e.g. in pipes and towers, when ground deformation produces strain and tilts) and thus the safety during the operations, or groundwater contamination affecting the local population. Thus, the characterization of both, subsurface and surface phenomena, is necessary to prevent potential problems.

These examples are in line with previous works available in the literature and confirm the need for improvement of the hydrocarbon industry and the whole society to properly prevent induced geohazards and their consequences. In our study case, potential damages caused by hydrocarbon productions activities, such as ground deformation and induced seismicity, are not being correctly assessed due to the absence of prevention policies. For example, both sites of this study are considered non seismic by the INPRES national seismic hazard zonification^29^ due to the absence of seismic events in historical terms. This means that the construction normative does not consider anti-seismic criteria in the area (even for the structures related to the new wells). In the GSJ basin the situation seems less alarming as only one event has been registered. However, the almost absolute absence of a scientific and public debate about the link between the industry and the earthquakes and its consequences, in the context of our two study sites, risks reproducing and aggravating the experiences lived in the last few years.

## Methods

This section describes the dataset and methods used in this work.

### SAR datasets

In this work have been used 4 different independent Sentinel-1 IW SAR datasets. One for each trajectory of the satellite (ascending and descending) for each test site. The Neuquén basin ascending dataset consists of 119 SLC images acquired between January 2017 and December 2020. The descending dataset consisted of 90 images covering the same period. The list of images of both trajectories is shown in Tables S6 and S7.

The GSJ basin dataset consisted in 104 descending images acquired between January 2017 and December 2020 and 91 ascending images covering the same period. The main earthquake event in the area is within the covered periods. The tables showing the full list of images are in the supplementary material (Tables S8 and S9).

### InSAR processing

The processing of the Sentinel-1 data have been carried out using the SBAS approach of the PSIG chain developed by the CTTC and^21,22^.

The main results provided by the approach are the displacement velocity maps and the temporal behaviour of each measured PS. The temporal coherence threshold to select PSs is set to 0.7. The images are multi-looked before starting the interferometric processing. We have used a pixel resolution of 30 by 30 m approximately. The main figures of the results are discussed in Sect. 2.

Finally, the coseismic interferograms have been processed by the CTTC interferometric processor. The interferograms have been unwrapped using the unwrapping approach described in^42^ We calculated an average interferogram for each earthquake, in ascending and descending geometry, averaging 11 coseismic ascending interferograms and 10 coseismic descending interferograms for the M_L_ 4.9, 2019 March 7 Neuquén basin earthquake and 4 coseismic ascending interferograms and 6 coseismic descending interferograms for the M_L_ 5, 2019 October 17 GSJ basin earthquake. See the supplementary material for details.

### Hydrocarbon production and injection volumes

The wells location and production data were downloaded from official public databases from the Argentine Energy Secretariat. The database provides an accurate description of the wells and information about owner company, well depth, well status (abandoned, operative), etc. Monthly production disaggregated information is reported for each well and fluid type (natural gas, oil, water). The same happens for the hydraulic fracturing process: injected materials, operating pressure and period, fracture stages quantity, horizontal branch length and some other information is provided.

All production quantities are given in cubic metres (m^3^), except injected sands and produced gas. Sands are given in metric tonnes that have been converted to m^3^ to allow comparisons. Gas is informed in thousands of m^3^ in standard conditions (1 bar and 288 K). We have converted them to equivalent m^3^ at reservoir conditions, applying PVT essential relations and the corresponding compressibility factor. This transformation allowed us to approximate the actual displaced volume from the geological formation. Oil and water volumes need no conversion. In the Neuquén basin, the studied wells are almost exclusively gas producers and gas is extracted around 3000 m beneath the surface, so determining the correct gas conversion factor is essential to understand the processes. Assuming typical pressure and temperature values for the formation we equated 1000 m^3^ of gas in standard conditions to 2 m^3^ beneath the surface. In the GSJ basin case (black oil), gas production has a negligible contribution, so a coarse approximation is valid for the analysis. Considering the scarce information about the physical conditions in the GSJ formations, we applied the same gas volume factor as in the Neuquén basin. In the GSJ basin, the variable truly controlling the production trends is water injection and extraction, and secondly, oil production, which in the area is around 10 times smaller than the water one.

We have scaled the production data to ease the comparison between ground deformation [mm] and production processes [m^3^]. Depending on a) the type of activity (injection or extraction, conventional or not), b) the number of involved wells in the displacement phenomena and c) the extension of the affected area, the amounts of fluid causing ground deformation can be considerably diverse. So, referring to the total nominal amount may mislead when observing different cases. For example, in Fig. 7 a balance volume in panel (b) which is more than two times bigger than in panel (d) produces equivalent deformation. This is explained because of the size of each affected area and the number of wells covering it (the density of wells by area unit and the total area size). Consequently, we standardised the production data. First, monthly amounts have been represented as a fraction of the total accumulated for the study period, as a per one ratio. Then, the curve has been scaled to fit the same numeric scale as ground deformation (we multiplied the monthly normalised production rate by the average of the accumulated ascending and descending deformation of each case and period). In the case of the subsidence, zones 1 and 2 of the Neuquén basin where the process is linked to the gas extraction, the curve has been inverted. All the data processed is available in the supplementary material.

### Seismic data

Both regions are deficiently covered by a seismic network, only few seismographs are sited hundreds of kilometres away of the study areas. This could explain the low b-value obtained from the seismic dataset (see supplementary data). Magnitude of completeness Mc is 2.4 M_L_ (supplementary figure S3), with an associated error of around 20 km in epicentre and hypocentre determination. In the Neuquén basin, before 2018, only a M_L_ 4.5 event in 2015 and a M_L_ 4.3 event in 2017 were reported, and more than two hundred since 2018. In the GSJ basin, apart from the M_L_ 5 2019 October 17th earthquake studied here, no seismic events were reported. The statements above are independent of the magnitude or historic period in both study areas^29^. To better adjust to scientific standards, the data corresponding to the most important events (M_L_ > 4) have been taken from the USGS database. Nevertheless, the seismic catalogue can be considered complete for earthquakes magnitudes M_L_ > 4.5 from the second half of the twentieth century attending to international agencies as USGS and ISC and historic reports.

### Modelling coseismic deformation

The source modelling was performed using the freely available Geodetic Bayesian Inversion Software (GBIS)^43^, which allows the estimation of the optimal source model parameters using a Bayesian approach for the inversion of multiple geodetic data sets. The inversion was carried out using a rectangular dislocation source within an elastic half-space^44^, with nine source model parameters (length, width, depth of the upper edge, dip angle, strike, X and Y coordinates of the midpoint of the upper edge, slip in the strike direction and uniform slip in the dip direction).

The GBIS software characterised the errors in each independent InSAR data set, such as randomly distributed noise and spatially correlated phase delays, by estimating variance and covariance in non-deforming areas. A linear ramp was also estimated during the inversion to remove any residual orbital error or very long wavelength atmospheric delay across the entire InSAR data sets.

The InSAR data subsampling carried out in GBIS, which uses a quadtree algorithm, resulted in 142 points for the Neuquén basin ascending data (from 83.611 initial points); 136 points for the Neuquén basin descending data (from 83.302 initial points), 176 points for the GSJ basin ascending data (from 128.546 initial points) and 214 points for the GSJ basin descending data (from 164.113 initial points). See Supplementary Figs. S7 and S16.

The results for the optimal nine fault source parameters and associated uncertainties in each case were obtained after 10^6^ iterations. The optimal parameters are extracted from the posterior probability density functions by finding the maximum a posteriori probability solution.

### Modelling deformation related to hydrocarbon production

These models were also performed using the Geodetic Bayesian Inversion Software (GBIS)^43^. In this case, the inversion was carried out using a point source within an isotropic elastic half-space^45^ with 4 source model parameters (depth of the point source, X and Y coordinates of the point source, volume change).

For the Neuquén basin, an area of ~ 20 km × 13 km containing the two areas of ground deformation (labelled as 1 and 2 in Fig. 2) was used for the inversion. The InSAR data subsampling resulted in 327 points for the Neuquén ascending data (from 151.837 initial points) and 76 points for the Neuquén basin descending data (from 188.204 initial points). See Supplementary Figs. S4 and S6.

For the GSJ basin, we modelled ground deformation zone labelled 3 in Fig. 6, contained in an area of ~ 12.5 × 8.5 km, which is only covered by our ascending data. The subsampling of these data resulted in 129 points (from 18.968 initial points). See Supplementary Figs. S13 and S15.

The results for the optimal point source parameters and associated uncertainties in each case were obtained after 10^6^ iterations. The optimal parameters are extracted from the posterior probability density functions by finding the maximum a posteriori probability solution.

## Supplementary Information

Supplementary Information.

## Data Availability

Public Argentinian hydrocarbon production reports and wells specifications are freely on-line available at the official webpage: https://datos.gob.ar/dataset/energia-produccion-petroleo-gas-por-pozo-capitulo-iv. Sentinel 1 ESA SAR Satellite images are freely on-line available at the official site: https://scihub.copernicus.eu/dhus/. Processed datasets derived from these raw data are open. Authors can share under request. Please write Corresponding author for any request.
