# Author Correction: Assessment of ground deformation and seismicity in two areas of intense hydrocarbon production in the Argentinian Patagonia

**DOI:** 10.1038/s41598-022-26998-y

**Published:** 2023-01-06

**Authors:** Guillermo Tamburini‑Beliveau, Javier A. Grosso‑Heredia, Marta Béjar‑Pizarro, Raúl Pérez‑López, Juan Portela, Martín Cismondi‑Duarte, Oriol Monserrat

**Affiliations:** 1 Centro de Investigaciones y Transferencia de Santa Cruz (CIT SC - CONICET), Lisandro de La Torre 860, Río Gallegos, Argentina; 2 Departamento de Geografía, Universidad del Comahue, Buenos Aires, 1400 Neuquén Argentina; 3 Instituto Geológico y Minero de España (IGME-CSIC), Rios Rosas, 23, 28003 Madrid, Spain; 4 Universidad Politécnica de Madrid. GI-Terra: Geomática, Amenazas Naturales y Riesgos, Mercator 2, 28031 Madrid, Spain; 5 Instituto de Investigación y Desarrollo en Ingeniería de Procesos y Química Aplicada (IPQA‑CONICET‑UNC), Ciudad Universitaria, Córdoba, Argentina; 6 Departament de Geomàtica, Centre Tecnològic de Telecomunicacions de Catalunya, Carl Friedrich Gauss 7, Castelldefels, Spain

Correction to: *Scientific Reports* 10.1038/s41598-022-23160-6, published online 10 November 2022

The original version of this Article contained errors.

In the Results section, under the subheading ‘The Neuquén basin’,

“Red tones represent points moving toward the satellite while blue ones show points moving far from the satellite. Figure 2c and d show the vertical and west–east components of the displacement. These components are derived from the LOS results following the approach described in 23. Red tones represent movements up and towards the east in the vertical and horizontal components respectively. It is worth noting the quality of this components rely on the quality of the LOS results.”

Now reads:

“Red tones represent points moving far from the satellite while blue ones show points moving towards the satellite. Figure 2c and d show the vertical and west–east components of the displacement. These components are derived from the LOS results following the approach described in 23. Red tones represent movements down and towards the east in the vertical and horizontal components respectively. It is worth noting the quality of these components rely on the quality of the LOS results.”

In the Results section, under the subheading ‘Hydrocarbon production and ground displacements in the GSJ basin’,

“The average displacement time series estimated for zones 1, 2 and 3 using the ascending, descending and vertical trajectories (orange and blue lines, respectively) are shown in Fig. 7b–d.”

Now reads:

The average displacement time series estimated for zones 1, 2 and 3 using the ascending, descending and vertical trajectories (orange, blue and gray dots, respectively) are shown in Fig. 7b–d.

The original Article has been corrected.

