# Supplementary material for: Assessment of ground deformation and seismicity in two areas of intense hydrocarbon production in the Argentinian Patagonia
Source: Sci Rep. 2022 Nov 10;12:19198. doi: 10.1038/s41598-022-23160-6 (PMC9649598; doi:10.1038/s41598-022-23160-6)
Supplement: Supplementary file 1 — Supplementary Information. [file 41598_2022_23160_MOESM1_ESM.pdf]

SUPPLEMENTARY MATERIAL

Assessment of ground deformation and seismicity associated to fluid injection and oil/gas extraction in the Argentinian Patagonia

Guillermo Tamburini-Beliveau[1], Javier A. Grosso-Heredia[2], Marta Béjar-Pizarro[3], Raúl Pérez-López[3], Juan Portela[4], Martín Cismondi-Duarte[5], Oriol Monserrat[6]

1.Centro de Investigaciones y Transferencia de Santa Cruz (CIT SC - CONICET), Lisandro de la Torre 860, Río Gallegos, Argentina.

2. Universidad del Comahue, Departamento de Geografía, Buenos Aires 1400, Neuquén, Argentina.

3. Geological Survey of Spain (CN IGME,CSIC), Rios Rosas, 23, Madrid 28003, Spain

4. Universidad Politécnica de Madrid, RG Terra: Geomatics, Natural Hazards and Risks, Mercator 2, 28031 Madrid, Spain.

5. Instituto de Investigación y Desarrollo en Ingeniería de Procesos y Química Aplicada (IPQA-CONICET-UNC). Ciudad Universitaria. Córdoba, Argentina.

6. Centre Tecnològic de Telecomunicacions de Catalunya, Departament de Geomàtica, Carl Friedrich Gauss 7, Castelldefels, Spain.

Supplementary Figure S1: Map of the distribution of the 45 stations of the national seismic network of the INPRES. The study areas are shown as bigger circles. The four nearest stations and their distance to each area have been detailed. Our own elaboration form source: http://contenidos.inpres.gob.ar/est_sism. The background is a DEM courtesy of National Oceanic and Atmospheric Administration (https://www.ngdc.noaa.gov/mgg/topo/gltiles.html).

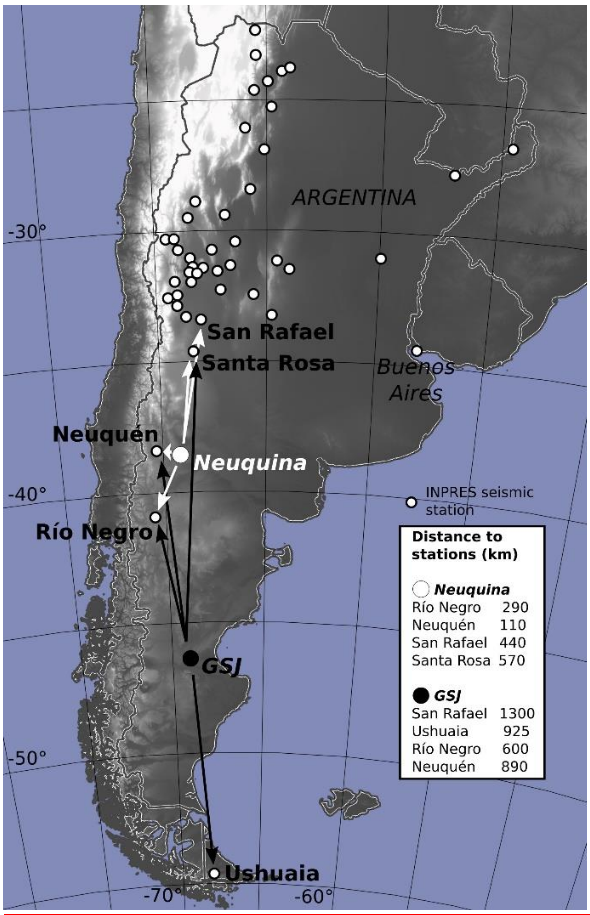

Supplementary Figure S2: A. Relationship between the injected volume and maximum seismic energy released from the Hike data base and Vaca Muerta (Neuquén) oil field. B. Relationship of total injected volume and maximum seismic in fracking cases. (Wilson et al., 2017).

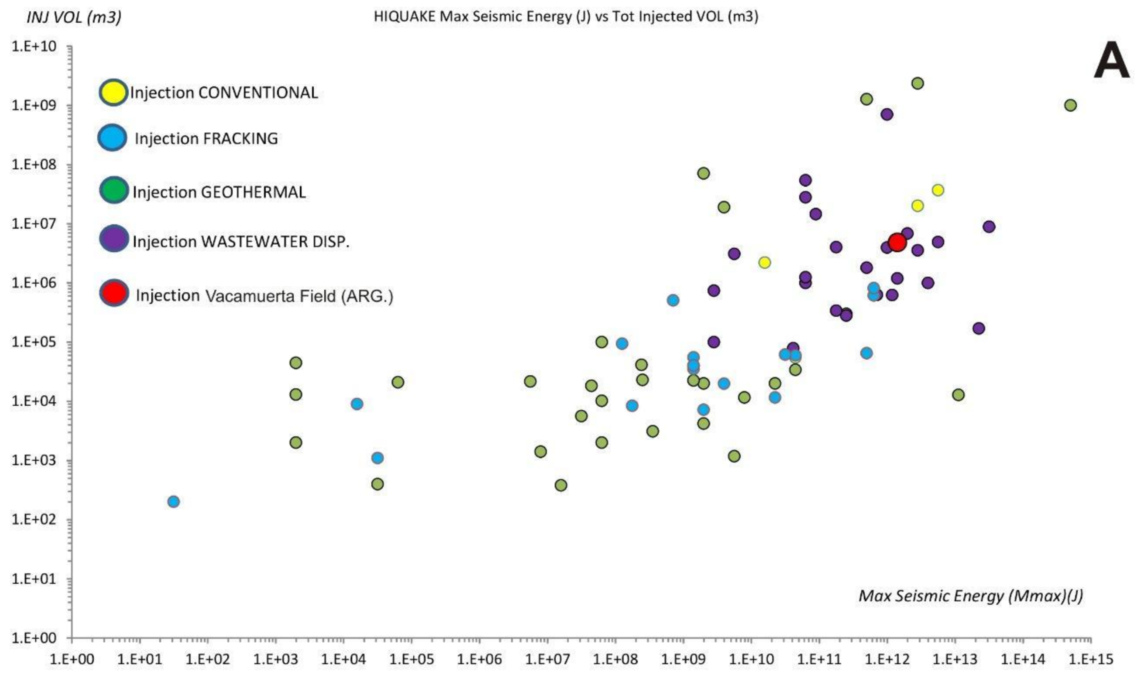

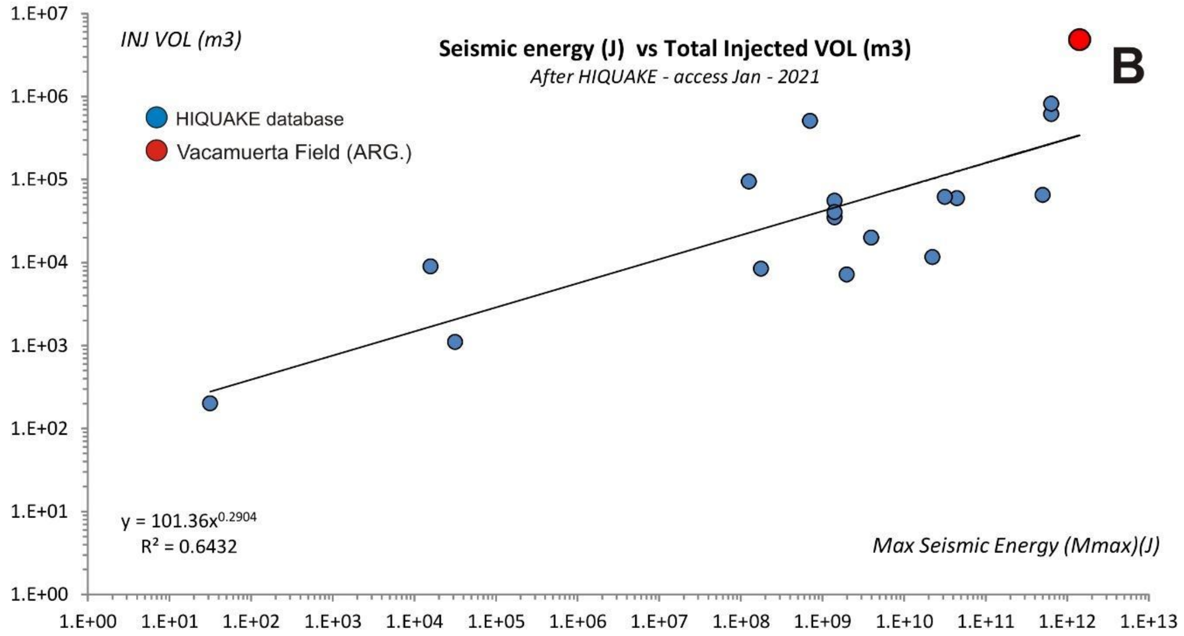

Supplementary Figure S3: A. Gutenberg and Richter law of the seismicity in Vaca Muerta. B. Comparison of the b-value between different induced cases and Vaca Muerta. (After Mousavi et al., 2017).

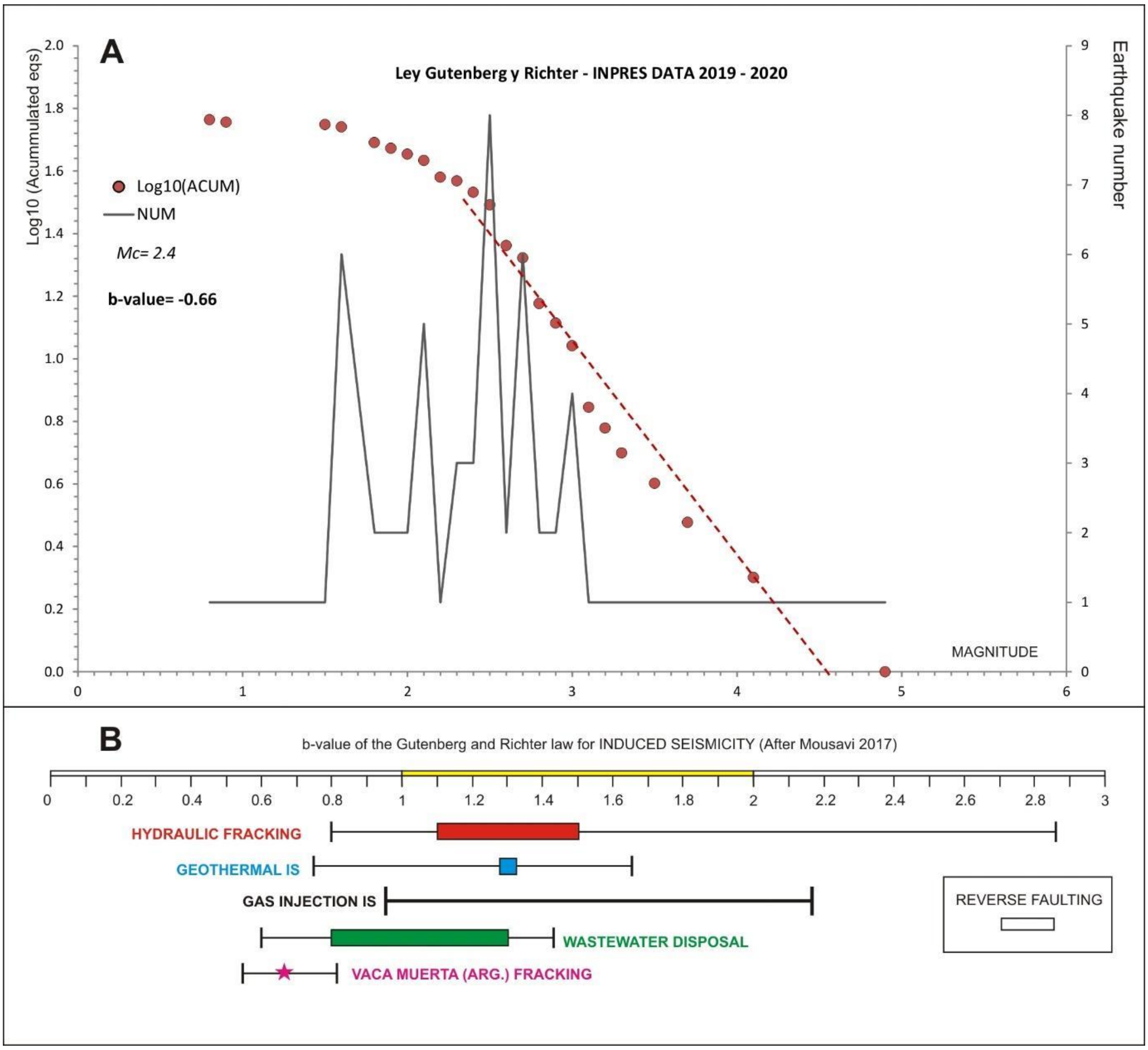

Supplementary Figure S4: GBIS quadtree subsampling for the accumulated ground deformation over the two deformation areas in Neuquén, previous to the Mogi modelling. Ascending (left) and descending (right).

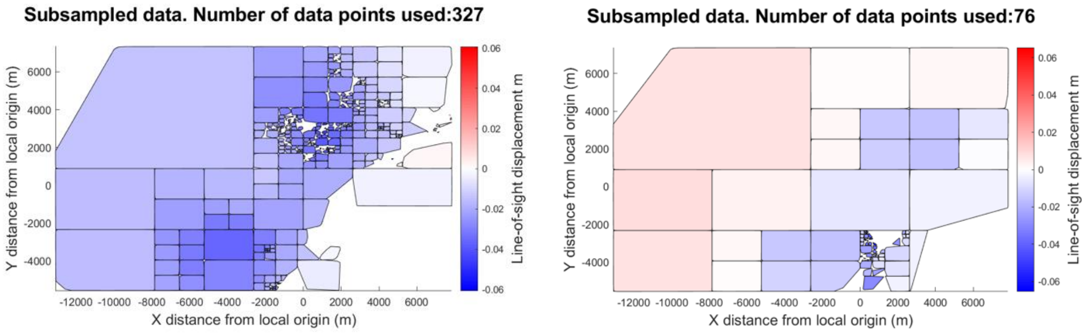

Supplementary Figure S5: GBIS inversion results for the Neuquén Mogi models: Posterior Probability density functions of the estimated parameters and the optimal parameter (red line).

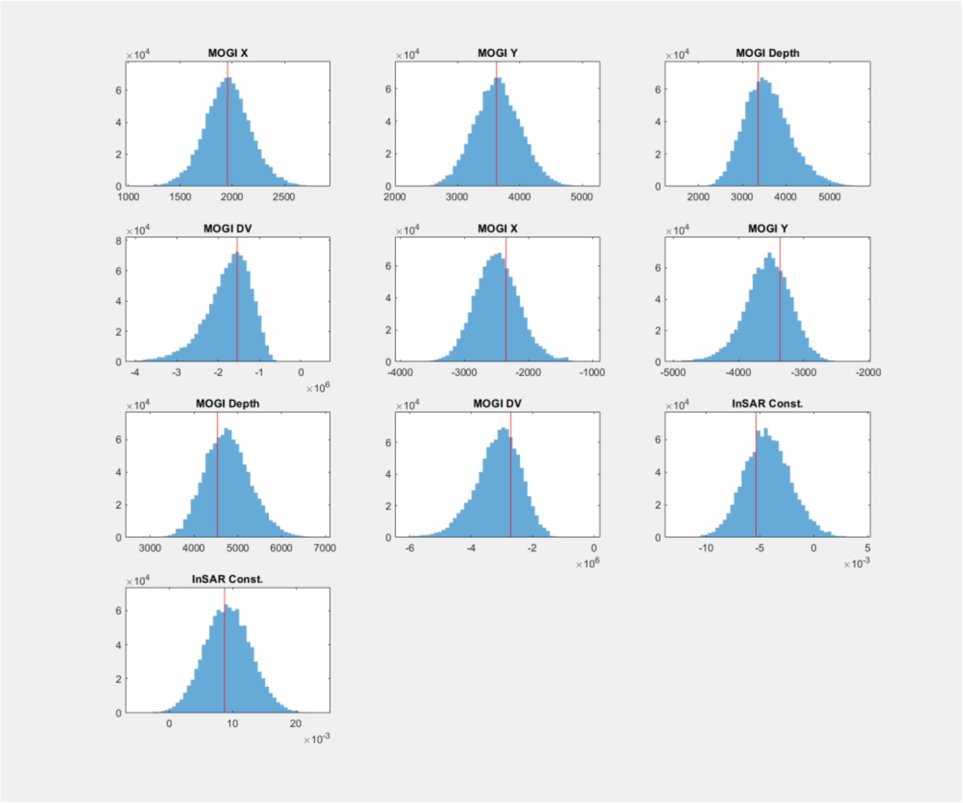

Supplementary Table S1: GBIS inversion results for the Neuquén Mogi model: Fitted parameters.

MODEL PARAM.	OPTIMAL	MEAN	Median	2.50%	97.50%
MOGI X	1952.94	1957.15	1954.06	1514.75	2411.96
MOGI Y	3623.65	3641.97	3635.87	2927.29	4390.05
MOGI Depth	3362.03	3577.81	3536.95	2648.85	4767.82
MOGI DV	-1.54E+06	-1.76E+06	-1.68E+06	-3.11E+06	-901403
MOGI X	-2351.89	-2481.97	-2494.6	-3144.9	-1730.33
MOGI Y	-3364.51	-3552.29	-3535.2	-4320.53	-2900.98
MOGI Depth	4531.65	4771.77	4748.6	3816.51	5879.74
MOGI DV	-2.71E+06	-3.14E+06	-3.07E+06	-4.77E+06	-1.84E+06
InSAR Const.	-0.005358	-0.004277	-0.004336	-0.008393	0.000159
InSAR Const.	0.008771	0.009284	0.009242	0.002227	0.016578

Supplementary Figure S6: (a) and (d) show accumulated LOS ground deformation over the two areas labelled 1 and 2 in figure 2a. (b) and (e) show LOS deformation predicted by the forward model using the maximum a posteriori probability solution. (c) and (f) show the residuals. The black crosses represent fracking wells and red triangles represent wastewater wells. The purple stars in (b) and (e) indicate the location of the Mogi sources.

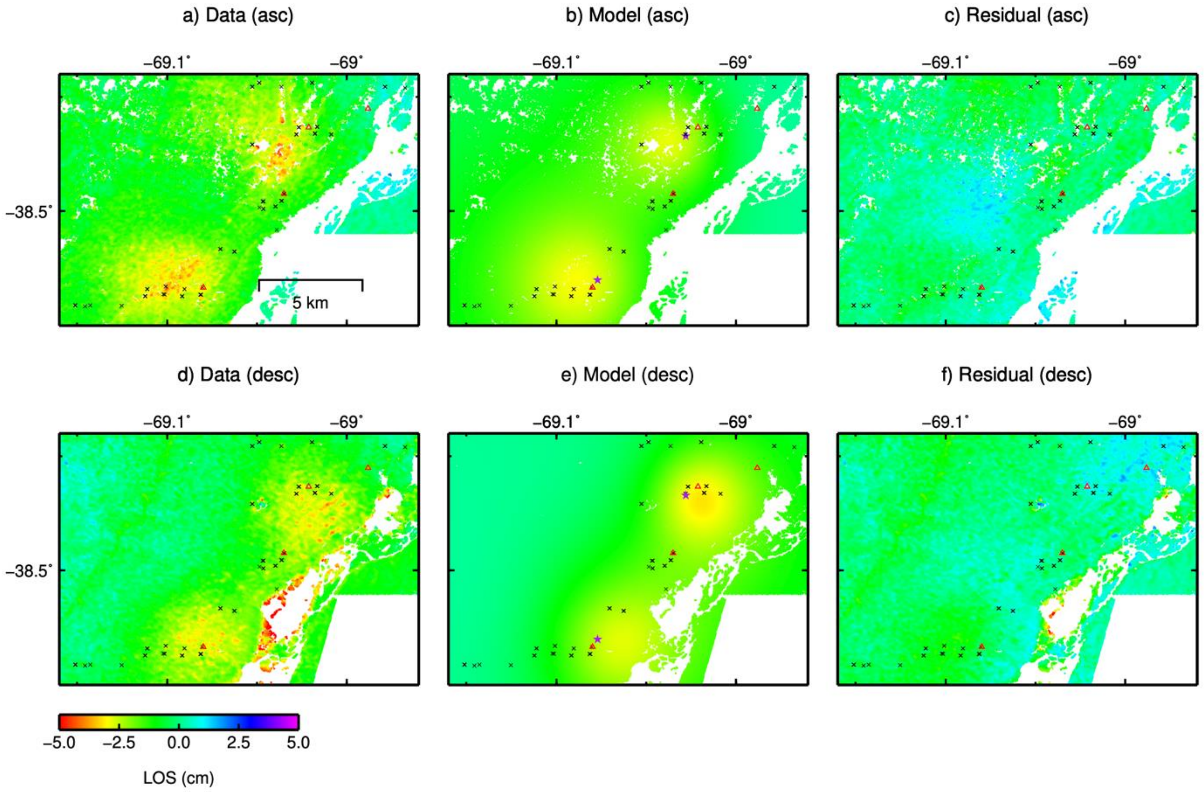

Supplementary Figure S7: GBIS (Bagnardi et al., 2018) quadtree subsampling for the averaged coseismic ascending (left) and descending (right) inteferograms in Neuquén.

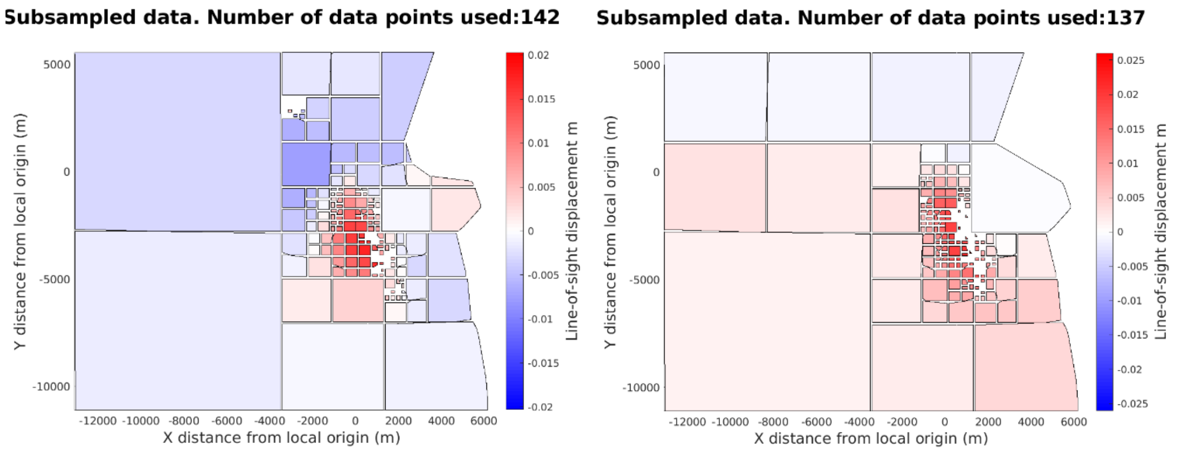

Supplementary Figure S8: GBIS (Bagnardi et al., 2018) inversion results for Neuquén dataset: Posterior Probability density functions of the estimated parameters and the optimal parameter (red line).

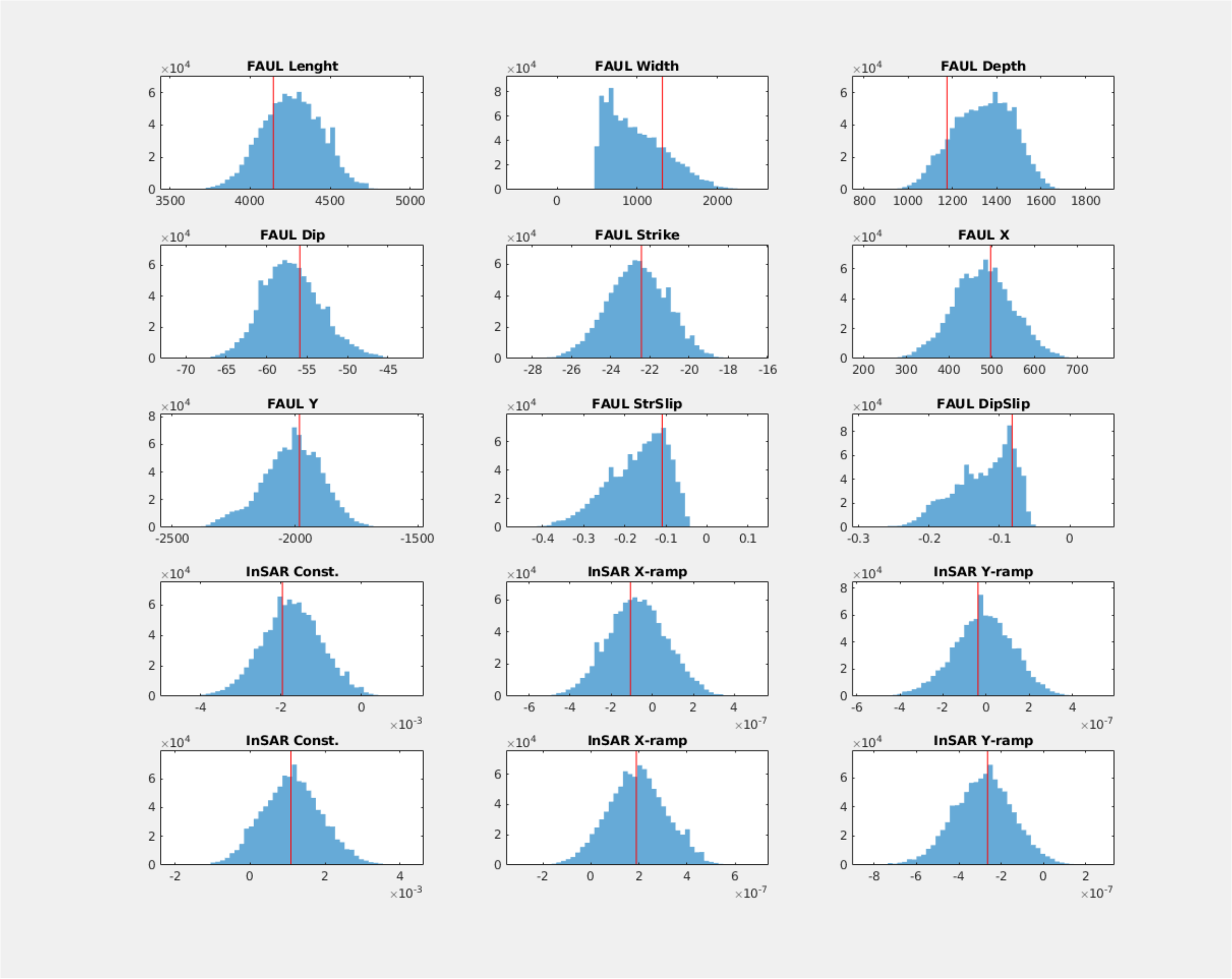

Supplementary Table S2: GBIS (Bagnardi et al., 2018) inversion results for Neuquén dataset: Fitted parameters.

MODEL_PARAM.	OPTIMAL	MEAN	Median	2.50%	97.50%
FAUL Lenght	4144.76	4261.05	4261.65	3894.99	4616.37
FAUL Width	1315.17	1001.66	931.657	516.153	1843.36
FAUL Depth	1174.76	1338.43	1345.71	1074.93	1577.11
FAUL Dip	-55.9039	-56.8738	-57.0763	-63.7028	-48.9589
FAUL Strike	-22.4351	-22.6457	-22.6421	-25.6695	-19.737
FAUL X	497.83	480.684	480.849	342.724	617.743
FAUL Y	-1982.3	-2012.86	-2003.91	-2277.66	-1789.68
FAUL StrSlip	-0.109332	-0.169608	-0.157119	-0.332249	-0.0611376
FAUL DipSlip	-0.0819114	-0.12287	-0.115147	-0.212325	-0.0639685
InSAR Const.	-1.96E-03	-1.73E-03	-1.73E-03	-3.21E-03	-2.73E-04
InSAR X-ramp	-1.06E-07	-7.27E-08	-7.44E-08	-3.51E-07	2.15E-07
InSAR Y-ramp	-3.75E-08	-1.35E-08	-1.35E-08	-2.96E-07	2.53E-07
InSAR Const.	1.09E-03	1.11E-03	1.12E-03	-4.05E-04	2.70E-03
InSAR X-ramp	1.89E-07	1.93E-07	1.93E-07	-4.81E-08	4.34E-07
InSAR Y-ramp	-2.64E-07	-2.85E-07	-2.80E-07	-5.66E-07	-1.06E-08

Supplementary Figure S9. Location of the 7 March 2019 earthquake (Neuquén basin), from this study (red star) and from other agencies (black stars in Los Barreales Reservoir). The background image is courtesy of Google Satellite images.

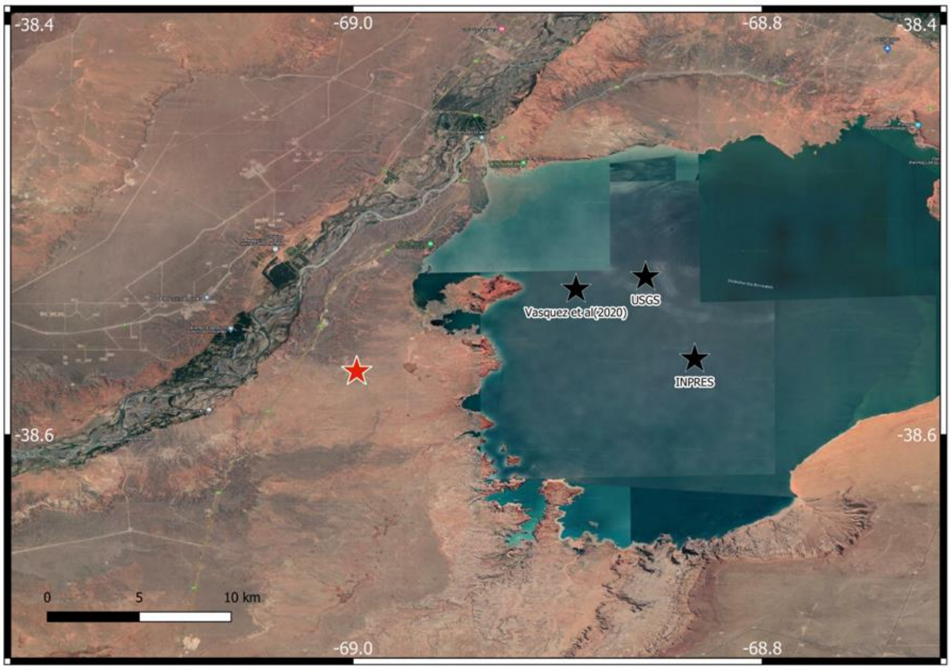

Supplementary Figure S10: Earthquakes located into the Vaca Muerta Basin with focal mechanism solutions for the November 19th 2015 earthquake (Correa-Otto et al. 2018) and the March 7th 2019 earthquake (from USGS). The Añelo Anticline structure controls the S_{Hmax} first order horizontal stress (N112°E, from this study).

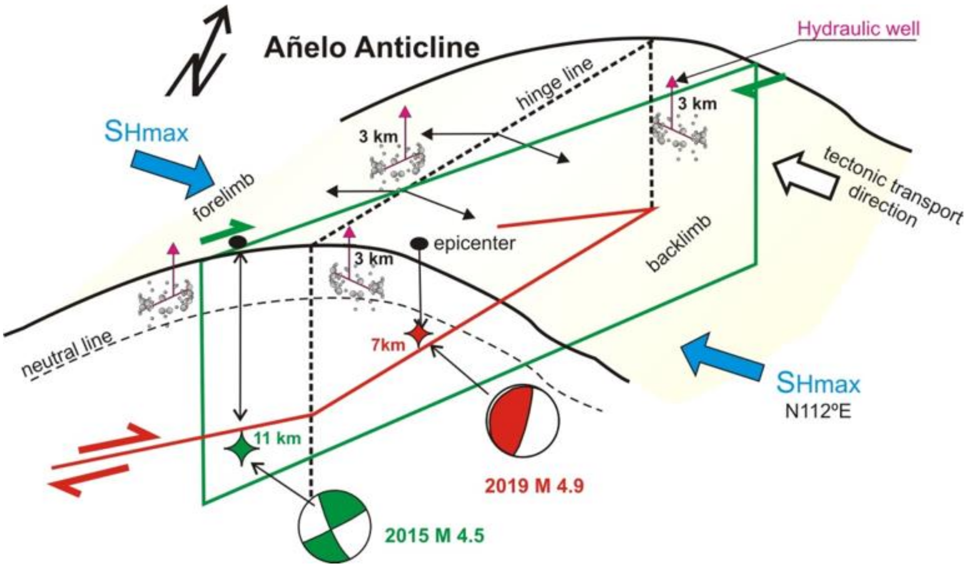

Supplementary Figure S11. Stress tensor solution obtained from Win-Tensor software (Delvaux and Sperner, 2003, downloaded at http://damiendelvaux.be/Tensor/WinTensor/win-tensor_download.html . S_{Hmax} is defined by 112°E ± 4° trend, with a tectonic compressive regime with strike slip component. Focal mechanism solutions were obtained from this study (upper), USGS (middle) and Correa-Otto et al. 2018 (bottom).

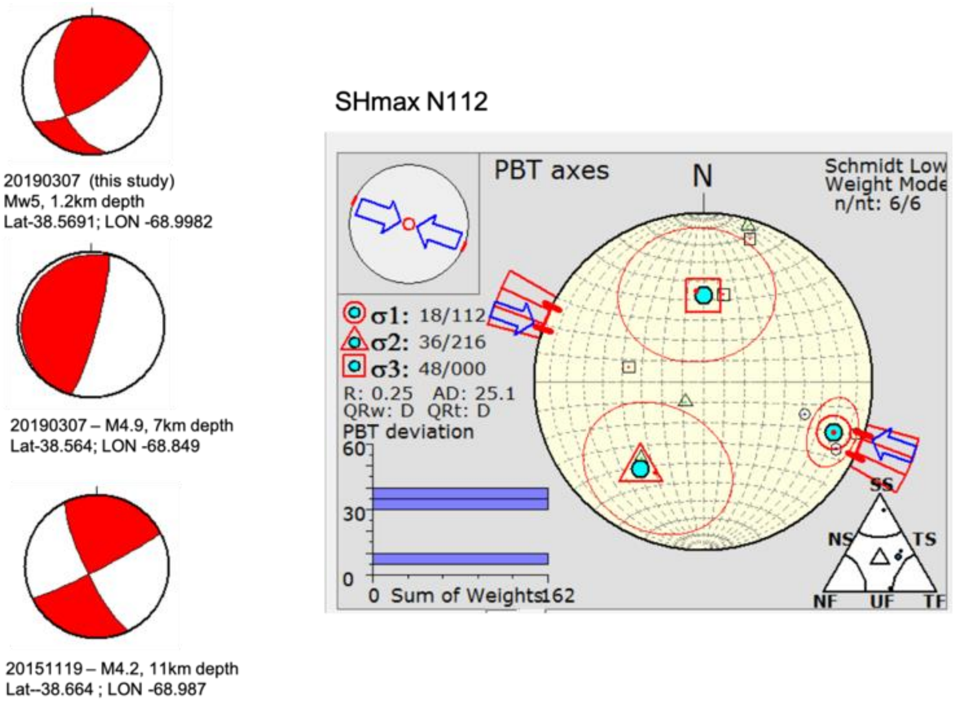

Supplementary Figure S12: The obtained stress direction (S_{Hmax} 112°E) is aligned with the subduction vector of the Nazca plate, and perpendicular to the topography. These local perturbations due to forces induced by local non meridian topographic axis or to basement controls lead to small rotations with respect to the general tectonic trend (S_{Hmax} N90°E). SH_{max} turns from N75°E northward to N117°E southward (Guzmán and Bottesi, 2007; Sanz-Perl and Delucci, 2017).

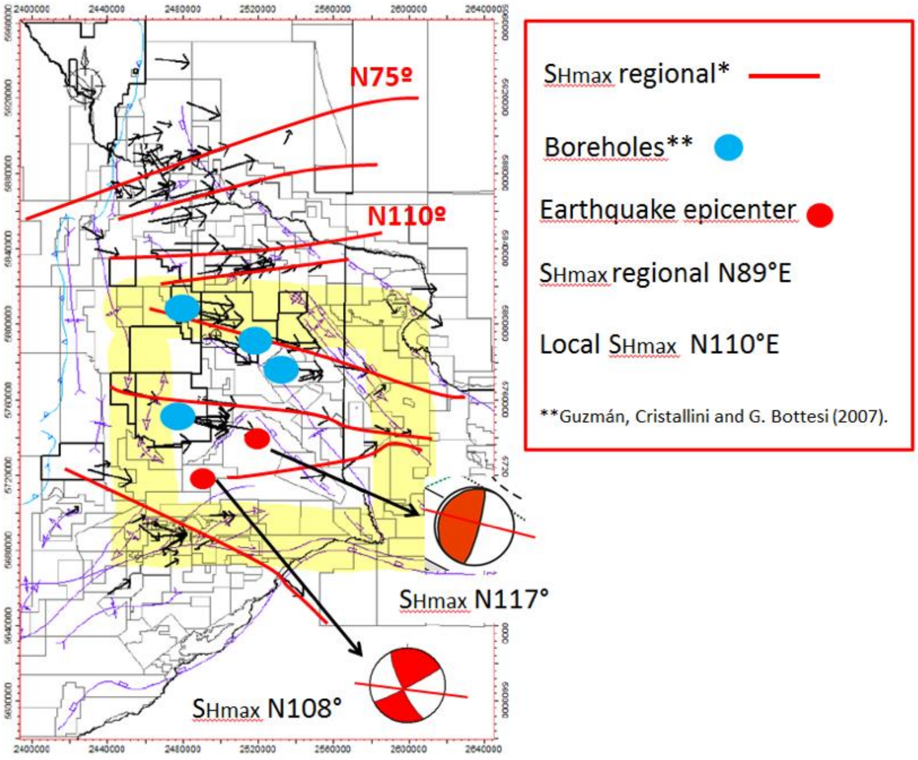

***Guzmán, Cristallini and G. Bottesi (2007).*

** Sanz Perl, D. Delucchi (2017)*

Supplementary Table S3: Values of S_{Hmax} estimated in this study and S_{Hmax} from previous studies (Guzman and Bottesi 2007, Sanz Perl and Delucchi 2017).

SHmax***	Tectonic reg.***	SHmax Regional	SHmax Local
N112°E	Reverse SS	N110°E* .N90°E**	N110°E* ESE**

** Sanz Perl, D. Delucchi (2017).*

***Guzmán et al. (2007).*

**** This study*

Supplementary Figure S13: GBIS quadtree subsampling for the accumulated ground deformation (ascending) over the deformation areas 3 in GSJ, previous to the Mogi modelling.

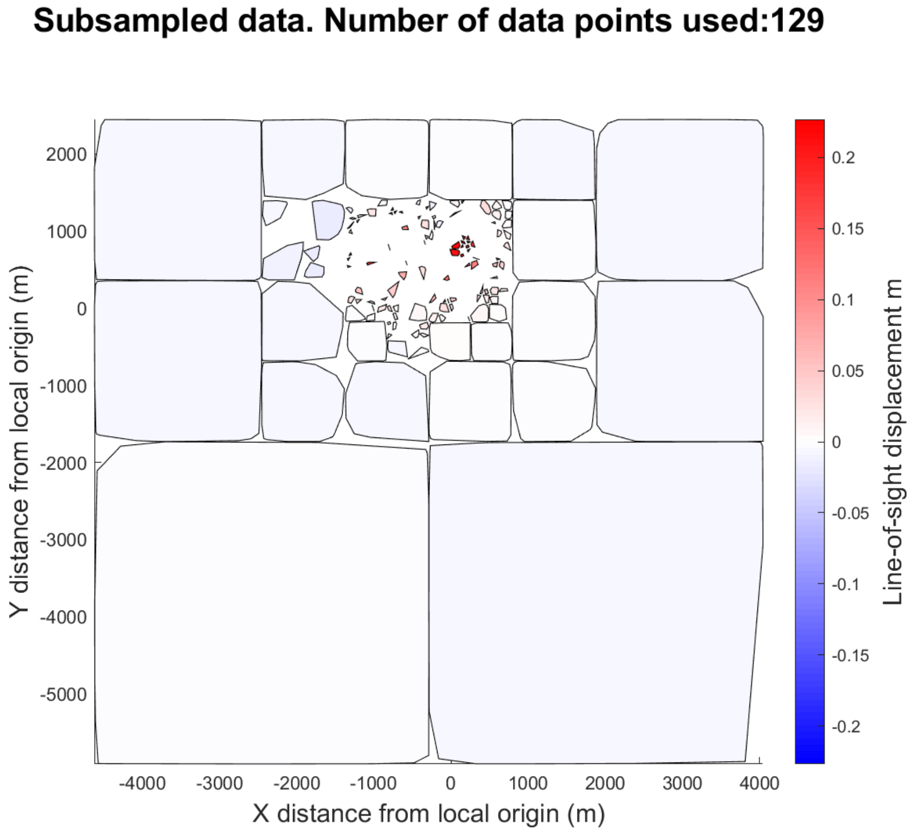

Supplementary Figure S14: GBIS inversion results for the GSJ Mogi models: Posterior Probability density functions of the estimated parameters and the optimal parameter (red line).

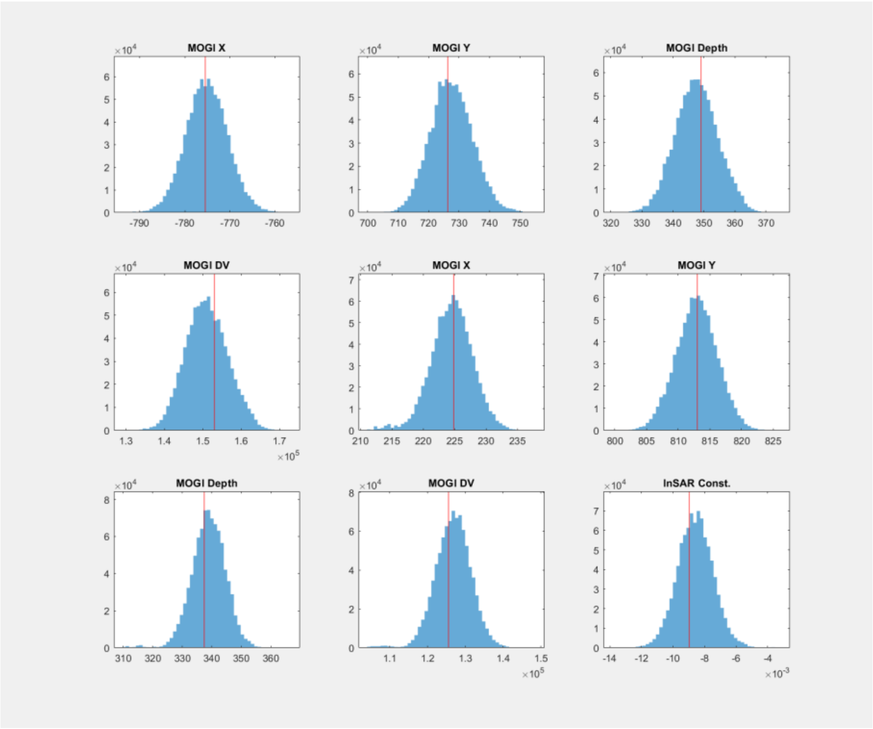

Supplementary Table S4: GBIS inversion results for the GSJ Mogi model: Fitted parameters.

MODEL PARAM.	OPTIMAL	MEAN	Median	2.50%	97.50%
MOGI X	-775.478	-775.111	-775.144	-784.063	-765.942
MOGI Y	726.254	727.546	727.361	714.484	741.353
MOGI Depth	349.072	347.678	347.623	334.454	361.078
MOGI DV	1.53E+05	1.51E+05	1.51E+05	1.41E+05	162081
MOGI X	224.827	224.515	224.628	217.58	230.89
MOGI Y	813.047	812.928	812.955	806.548	819.198
MOGI Depth	337.452	338.336	338.888	323.965	349.519
MOGI DV	1.26E+05	1.26E+05	1.27E+05	1.15E+05	1.36E+05
InSAR Const.	-0.008982	-0.008501	-0.008562	-0.010781	-0.005970

Supplementary Figure S15: Figure (a) shows accumulated LOS ground deformation over the area labelled 3 in figure 6a. Figure (b) shows LOS deformation predicted by the forward model using the maximum a posteriori probability solution. Figure (c) shows the residuals. The black crosses represent production wells. The two black stars in figure (b) indicate the location of the Mogi sources.

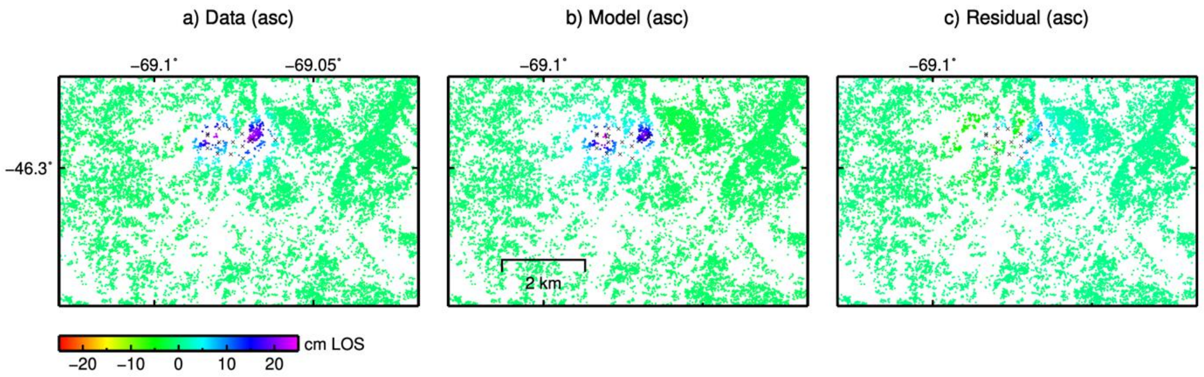

Supplementary Figure 16: GBIS (Bagnardi et al., 2018) quadtree subsampling for the averaged coseismic ascending (left) and descending (right) inteferograms in GSJ.

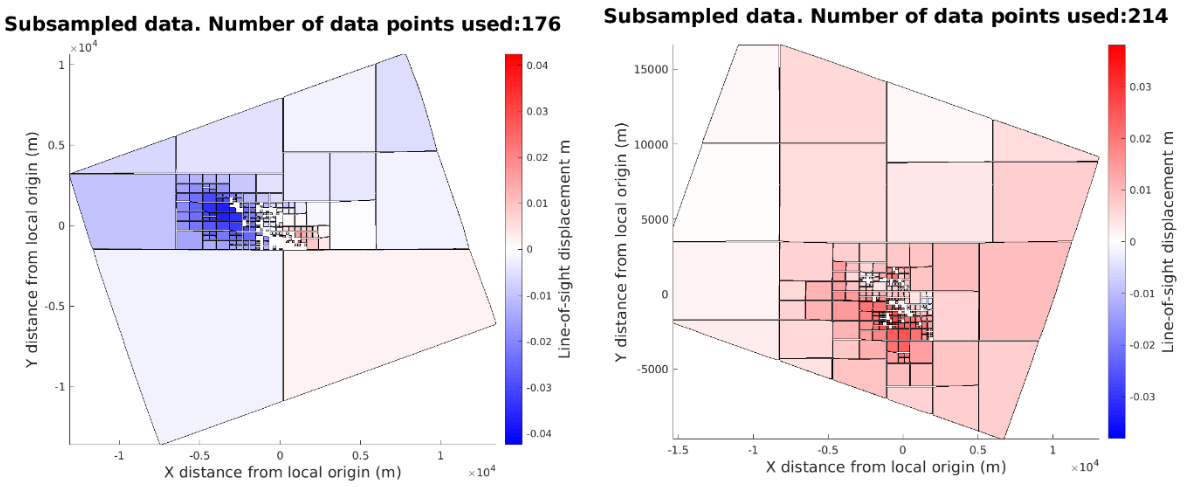

Supplementary Figure S17: GBIS (Bagnardi et al., 2018) inversion results for the GSJ dataset: Posterior Probability density functions of the estimated parameters and the optimal parameter (red line).

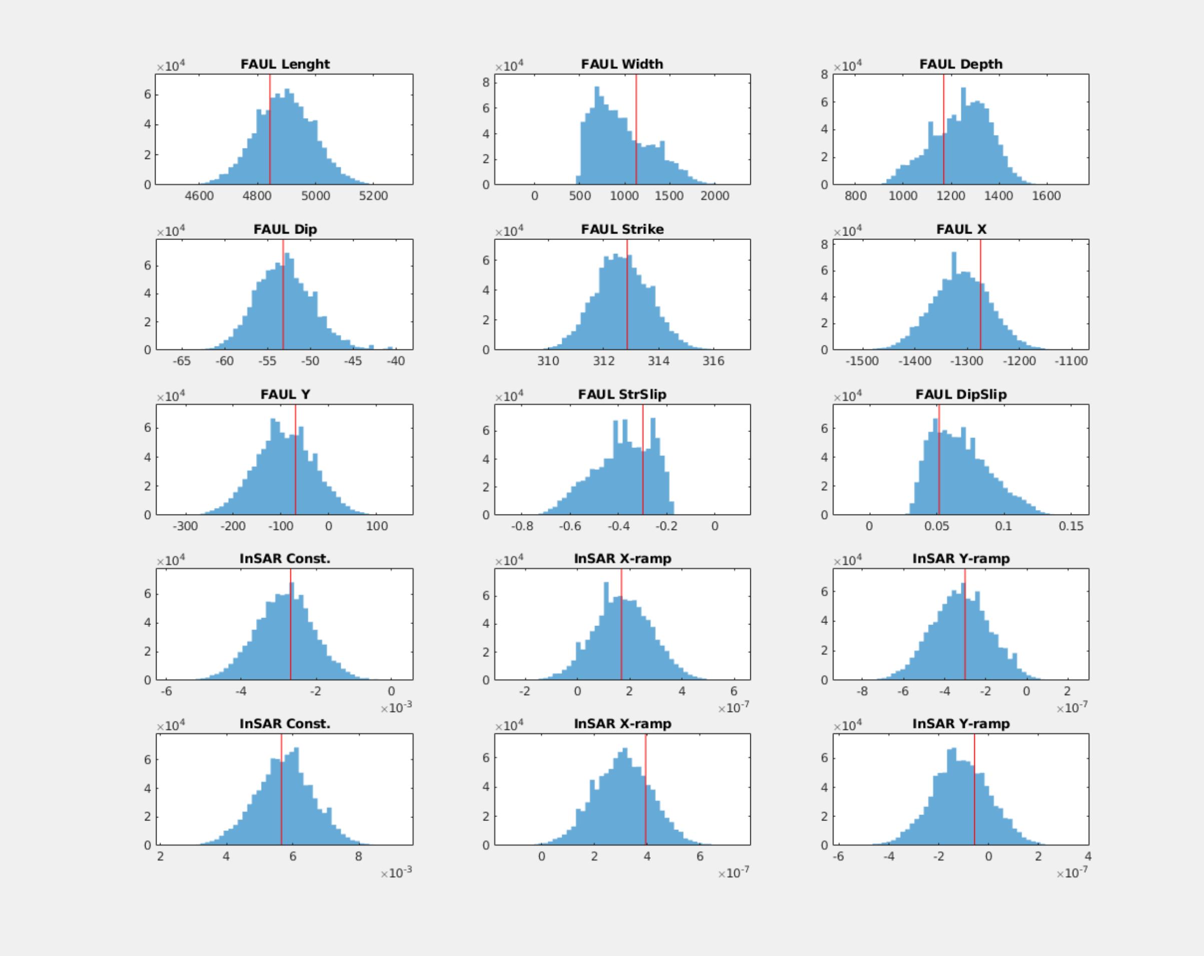

Supplementary Table S5: GBIS (Bagnardi et al., 2018) inversion results for the GSJ dataset: Fitted parameters.

MODEL_PARAM.	OPTIMAL	MEAN	Median	2.50%	97.50%
FAUL Lenght	4843.03	4893.55	4893.89	4696.59	5091.46
FAUL Width	1128.25	976.872	912.679	531.004	1675.75
FAUL Depth	1168.61	1240.94	1254.47	986.888	1450.74
FAUL Dip	-53.1814	-52.9998	-53.0878	-59.2783	-45.8194
FAUL Strike	312.866	312.699	312.675	310.758	314.7
FAUL X	-1274.65	-1311.93	-1312.51	-1421.32	-1202.92
FAUL Y	-69.5083	-92.2837	-93.435	-213.359	26.7808
FAUL StrSlip	-0.297806	-0.381692	-0.371297	-0.635518	-0.19788
FAUL DipSlip	0.0517062	0.0679784	0.0654109	0.0356889	0.116246
InSAR Const.	-2.69E-03	-2.85E-03	-2.83E-03	-4.39E-03	-1.32E-03
InSAR X-ramp	1.68E-07	1.72E-07	1.70E-07	-4.33E-08	3.92E-07
InSAR Y-ramp	-3.01E-07	-3.20E-07	-3.19E-07	-5.97E-07	-4.94E-08
InSAR Const.	5.66E-03	5.76E-03	5.78E-03	3.99E-03	7.47E-03
InSAR X-ramp	3.93E-07	3.06E-07	3.07E-07	9.10E-08	5.22E-07
InSAR Y-ramp	-5.73E-08	-1.11E-07	-1.14E-07	-3.40E-07	1.17E-07

Supplementary Figure S18. Location of the 17 October 2019 earthquake (GSJ basin), from this study (red star) and from other agencies (black stars). The background image is courtesy of Google Satellite images.

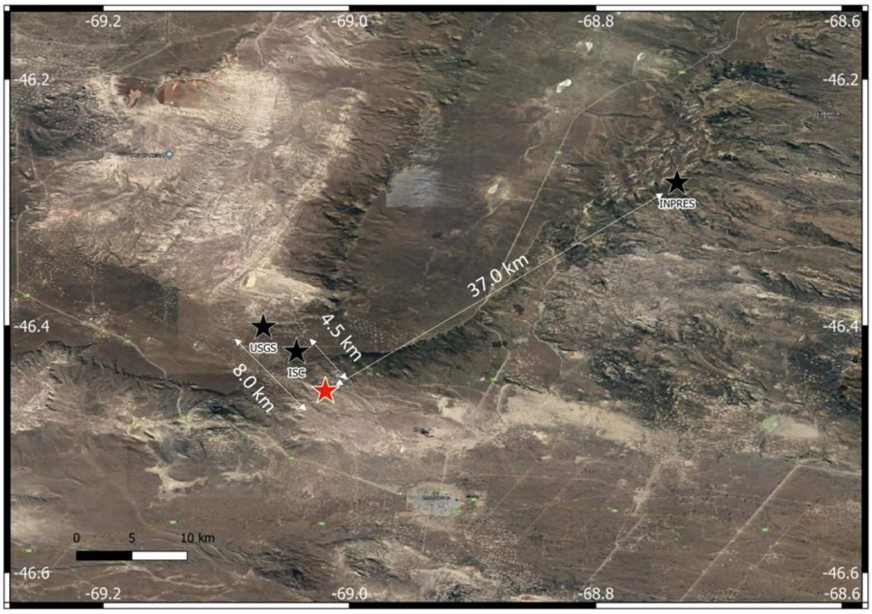

Supplementary Table S6: PS image dataset Neuquén 01/2017-12/2020 - Descending trajectory.

Neuqen Descending SAR images							
1	05/01/2017	23	13/11/2017	45	07/05/2019	67	07/02/2020
2	29/01/2017	24	25/11/2017	46	19/05/2019	68	19/02/2020
3	22/02/2017	25	07/12/2017	47	31/05/2019	69	02/03/2020
4	06/03/2017	26	17/02/2018	48	12/06/2019	70	14/03/2020
5	18/03/2017	27	01/03/2018	49	24/06/2019	71	26/03/2020
6	30/03/2017	28	13/03/2018	50	06/07/2019	72	07/04/2020
7	11/04/2017	29	25/03/2018	51	18/07/2019	73	01/05/2020
8	23/04/2017	30	27/10/2018	52	30/07/2019	74	13/05/2020
9	05/05/2017	31	08/11/2018	53	11/08/2019	75	25/05/2020
10	17/05/2017	32	20/11/2018	54	23/08/2019	76	12/07/2020
11	29/05/2017	33	02/12/2018	55	04/09/2019	77	05/08/2020
12	10/06/2017	34	14/12/2018	56	16/09/2019	78	17/08/2020
13	22/06/2017	35	26/12/2018	57	28/09/2019	79	29/08/2020
14	04/07/2017	36	07/01/2019	58	10/10/2019	80	10/09/2020
15	16/07/2017	37	19/01/2019	59	22/10/2019	81	16/10/2020
16	28/07/2017	38	31/01/2019	60	03/11/2019	82	28/10/2020
17	09/08/2017	39	12/02/2019	61	15/11/2019	83	09/11/2020
18	21/08/2017	40	24/02/2019	62	27/11/2019	84	21/11/2020
19	02/09/2017	41	08/03/2019	63	09/12/2019	85	03/12/2020
20	14/09/2017	42	20/03/2019	64	21/12/2019	86	15/12/2020
21	26/09/2017	43	01/04/2019	65	02/01/2020	87	27/12/2020
22	08/10/2017	44	13/04/2019	66	14/01/2020		
23	01/11/2017	45	25/04/2019	67	26/01/2020		

Supplementary Table S7: PS image dataset Neuquén 01/2017-12/2020 - Ascending trajectory.

Neuqen Ascending SAR images									
1	11/01/2017	23	25/03/2018	45	19/01/2019	67	22/09/2019	89	18/07/2020
2	17/05/2017	24	06/04/2018	46	31/01/2019	68	04/10/2019	90	30/07/2020
3	29/05/2017	25	18/04/2018	47	12/02/2019	69	16/10/2019	91	05/08/2020
4	10/06/2017	26	30/04/2018	48	24/02/2019	70	28/10/2019	92	11/08/2020
5	22/06/2017	27	12/05/2018	49	08/03/2019	71	21/11/2019	93	17/08/2020
6	04/07/2017	28	24/05/2018	50	20/03/2019	72	03/12/2019	94	23/08/2020
7	16/07/2017	29	05/06/2018	51	01/04/2019	73	15/12/2019	95	29/08/2020
8	28/07/2017	30	17/06/2018	52	13/04/2019	74	27/12/2019	96	04/09/2020
9	09/08/2017	31	11/07/2018	53	19/04/2019	75	08/01/2020	97	10/09/2020
10	21/08/2017	32	23/07/2018	54	25/04/2019	76	20/01/2020	98	16/09/2020
11	14/09/2017	33	04/08/2018	55	01/05/2019	77	01/02/2020	99	28/09/2020
12	26/09/2017	34	28/08/2018	56	13/05/2019	78	13/02/2020	100	04/10/2020
13	08/10/2017	35	09/09/2018	57	25/05/2019	79	08/03/2020	101	10/10/2020
14	20/10/2017	36	21/09/2018	58	31/05/2019	80	20/03/2020	102	16/10/2020
15	01/11/2017	37	03/10/2018	59	06/06/2019	81	01/04/2020	103	22/10/2020
16	13/11/2017	38	15/10/2018	60	18/06/2019	82	13/04/2020	104	03/11/2020
17	25/11/2017	39	27/10/2018	61	30/06/2019	83	25/04/2020	105	09/11/2020
18	07/12/2017	40	08/11/2018	62	12/07/2019	84	07/05/2020	106	15/11/2020
19	19/12/2017	41	20/11/2018	63	24/07/2019	85	19/05/2020	107	27/11/2020

20	31/12/2017	42	02/12/2018	64	05/08/2019	86	31/05/2020	108	09/12/2020
21	17/02/2018	43	14/12/2018	65	17/08/2019	87	12/06/2020	109	15/12/2020
22	01/03/2018	44	26/12/2018	66	29/08/2019	88	24/06/2020	110	21/12/2020
23	13/03/2018	45	07/01/2019	67	10/09/2019	89	06/07/2020	111	27/12/2020

Supplementary Table S8: PS image dataset GSJ 01/2017-12/2020 - Ascending trajectory.

GSJ Ascending SAR images									
1	21/01/2017	23	16/02/2019	45	27/08/2019	67	10/06/2020	89	19/12/2020
2	14/02/2017	24	22/02/2019	46	08/09/2019	68	16/06/2020	90	25/12/2020
3	20/07/2017	25	28/02/2019	47	02/10/2019	69	15/08/2020	91	31/12/2020
4	09/02/2018	26	06/03/2019	48	14/10/2019	70	21/08/2020		
5	21/02/2018	27	12/03/2019	49	26/10/2019	71	27/08/2020		
6	05/03/2018	28	18/03/2019	50	07/11/2019	72	02/09/2020		
7	31/10/2018	29	24/03/2019	51	19/11/2019	73	08/09/2020		
8	12/11/2018	30	30/03/2019	52	01/12/2019	74	14/09/2020		
9	18/11/2018	31	05/04/2019	53	13/12/2019	75	20/09/2020		
10	24/11/2018	32	11/04/2019	54	25/12/2019	76	26/09/2020		
11	30/11/2018	33	29/04/2019	55	06/01/2020	77	02/10/2020		
12	06/12/2018	34	05/05/2019	56	18/01/2020	78	08/10/2020		
13	12/12/2018	35	11/05/2019	57	30/01/2020	79	14/10/2020		
14	18/12/2018	36	17/05/2019	58	11/02/2020	80	20/10/2020		
15	24/12/2018	37	23/05/2019	59	23/02/2020	81	26/10/2020		
16	30/12/2018	38	29/05/2019	60	06/03/2020	82	01/11/2020		
17	05/01/2019	39	04/06/2019	61	18/03/2020	83	07/11/2020		
18	11/01/2019	40	16/06/2019	62	30/03/2020	84	13/11/2020		
19	17/01/2019	41	28/06/2019	63	11/04/2020	85	19/11/2020		
20	23/01/2019	42	10/07/2019	64	23/04/2020	86	25/11/2020		
21	29/01/2019	43	22/07/2019	65	05/05/2020	87	01/12/2020		
22	04/02/2019	44	03/08/2019	66	17/05/2020	88	07/12/2020		
23	10/02/2019	45	15/08/2019	67	29/05/2020	89	13/12/2020		

Supplementary Table S9: PS image dataset GSJ 01/2017-12/2020 – Descending trajectory.

GSJ Descending SAR images									
1	07/01/2017	23	03/11/2017	45	29/10/2018	67	01/08/2019	89	03/05/2020
2	31/01/2017	24	15/11/2017	46	10/11/2018	68	13/08/2019	90	15/05/2020
3	24/02/2017	25	27/11/2017	47	22/11/2018	69	25/08/2019	91	27/05/2020
4	08/03/2017	26	09/12/2017	48	04/12/2018	70	06/09/2019	92	08/06/2020
5	20/03/2017	27	21/12/2017	49	16/12/2018	71	18/09/2019	93	20/06/2020
6	01/04/2017	28	02/01/2018	50	28/12/2018	72	30/09/2019	94	19/08/2020
7	13/04/2017	29	14/01/2018	51	09/01/2019	73	12/10/2019	95	12/09/2020
8	25/04/2017	30	26/01/2018	52	21/01/2019	74	24/10/2019	96	24/09/2020
9	07/05/2017	31	07/02/2018	53	02/02/2019	75	05/11/2019	97	06/10/2020
10	19/05/2017	32	19/02/2018	54	14/02/2019	76	17/11/2019	98	18/10/2020
11	31/05/2017	33	03/03/2018	55	26/02/2019	77	29/11/2019	99	30/10/2020
12	12/06/2017	34	15/03/2018	56	10/03/2019	78	11/12/2019	100	11/11/2020

13	24/06/2017	35	08/04/2018	57	22/03/2019	79	23/12/2019	101	23/11/2020
14	06/07/2017	36	20/04/2018	58	03/04/2019	80	04/01/2020	102	05/12/2020
15	18/07/2017	37	02/05/2018	59	15/04/2019	81	16/01/2020	103	17/12/2020
16	30/07/2017	38	14/05/2018	60	27/04/2019	82	28/01/2020	104	29/12/2020
17	11/08/2017	39	26/05/2018	61	09/05/2019	83	09/02/2020		
18	23/08/2017	40	07/06/2018	62	21/05/2019	84	21/02/2020		
19	04/09/2017	41	19/06/2018	63	02/06/2019	85	04/03/2020		
20	16/09/2017	42	01/07/2018	64	14/06/2019	86	16/03/2020		
21	28/09/2017	43	25/07/2018	65	26/06/2019	87	28/03/2020		
22	10/10/2017	44	06/08/2018	66	08/07/2019	88	09/04/2020		
23	22/10/2017	45	17/10/2018	67	20/07/2019	89	21/04/2020		

Supplementary Files 1: We also attach the zip file 01_supplement.zip that contains all collected and used production data.

REFERENCES

Bagnardi, M., & Hooper, A. (2018). Inversion of surface deformation data for rapid estimates of source parameters and uncertainties: A Bayesian approach. Geochemistry, Geophysics, Geosystems, 19, 2194–2211. https://doi.org/10.1029/ 2018GC007585

Correa-Otto, S., S. Nacif, A. Pesce, A. Nacif, G. Gianni, R. Furlani, M. Giménez, R. Francisco, 2018. Intraplate seismicity recorded by a local network in the Neuquén Basin, Argentina, Journal of South American Earth Sciences, 87 211-220, https://doi.org/10.1016/j.jsames.2017.12.007

Delvaux, D., Sperner, B., 2003. New aspects of tectonic stress inversion with reference to the TENSOR program. Geol. Soc. Lond. Special Publ. 212, 75–100. https://doi.org/10.1144/GSL.SP.2003.212.01.06

Guzmán, Cristallini and G. Bottesi (2007). Contemporary stress orientations in the Andean retroarc. Tectonics vol. 26, TC3016, doi:10.1029/2006TC00195.Mousavi S. M., Ogwari P.O., Horton S.P., Langston C.A. 2017. Spatio-temporal evolution of frequency-magnitude distribution and seismogenic index during initiation of induced seismicity at Guy-Greenbrier, Arkansas, Physics of the Earth and Planetary Interiors, Volume 267, Pages 53-66, https://doi.org/10.1016/j.pepi.2017.04.005.

INPRES (2021a) INRPES. Buscador de sismos. http://contenidos.inpres.gob.ar/buscar_sismo

ISC (2021) International Seismological Centre. http://www.isc.ac.uk/iscbulletin/search/catalogue/

Mousavi, S. M., Ogwari, P. O., Horton, S. P. & Langston, C. A. (2017). Spatio-temporal evolution of frequency-magnitude distribution and seismogenic index during initiation of induced seismicity at Guy-Greenbrier, Arkansas. Phys. Earth Planet. Inter. 267, 53-66.

Sanz Perl, Y, Delucchi, D. (2017). Stress Organization along Neuquen Basin in Vaca Muerta Formation and their Impact in Microseismic Response. EAGE. Latin - American Seminar in Unconventional Resources 23 - 24 November 2017, Mexico City, Mexico. 4pp.

USGS (2021) Latest earthquakes. USGS. https://earthquake.usgs.gov/earthquakes/map/

Wilson M. P. , Foulger G. R., Gluyas J. G., Davies R. J., Julian B. R. 2017. HiQuake: The Human-Induced Earthquake Database. Seismological Research Letters, 88 (6): 1560–1565. doi: https://doi.org/10.1785/0220170112
